# A Computational Analysis of Neural Mechanisms Underlying the Maturation of Multisensory Speech Integration in Neurotypical Children and Those on the Autism Spectrum

**DOI:** 10.3389/fnhum.2017.00518

**Published:** 2017-10-30

**Authors:** Cristiano Cuppini, Mauro Ursino, Elisa Magosso, Lars A. Ross, John J. Foxe, Sophie Molholm

**Affiliations:** ^1^Department of Electric, Electronic and Information Engineering, University of Bologna, Bologna, Italy; ^2^Departments of Pediatrics and Neuroscience, Albert Einstein College of Medicine, Bronx, NY, United States; ^3^Department of Neuroscience and The Del Monte Institute for Neuroscience, University of Rochester School of Medicine, Rochester, NY, United States

**Keywords:** Hebbian learning rules, McGurk effect, development, multisensory training, neural network, speech comprehension

## Abstract

Failure to appropriately develop multisensory integration (MSI) of audiovisual speech may affect a child's ability to attain optimal communication. Studies have shown protracted development of MSI into late-childhood and identified deficits in MSI in children with an autism spectrum disorder (ASD). Currently, the neural basis of acquisition of this ability is not well understood. Here, we developed a computational model informed by neurophysiology to analyze possible mechanisms underlying MSI maturation, and its delayed development in ASD. The model posits that strengthening of feedforward and cross-sensory connections, responsible for the alignment of auditory and visual speech sound representations in posterior superior temporal gyrus/sulcus, can explain behavioral data on the acquisition of MSI. This was simulated by a training phase during which the network was exposed to unisensory and multisensory stimuli, and projections were crafted by Hebbian rules of potentiation and depression. In its mature architecture, the network also reproduced the well-known multisensory McGurk speech effect. Deficits in audiovisual speech perception in ASD were well accounted for by fewer multisensory exposures, compatible with a lack of attention, but not by reduced synaptic connectivity or synaptic plasticity.

## Introduction

As an organism interacts with its environment, objects and events stimulate its sundry sensory epithelia, providing oftentimes redundant and/or complementary cues to an object's presence, location, and identity. The ability to exploit these multiple cues is fundamental not just for optimized detection and localization of external events, but also for more demanding perceptual-cognitive tasks, such as those involved in communication. For example, the intelligibility of speech is significantly improved when one can see the speaker's accompanying articulations, a multisensory benefit that is readily demonstrated under noisy listening conditions (Sumby and Pollack, 1954; Ross et al., 2007) and one that clearly impacts the development of human communication. Multisensory integration (MSI) has a protracted developmental trajectory that appears to be highly immature at birth (Wallace and Stein, 1997; Lewkowicz et al., 2015) and that continues to develop late into childhood (Ross et al., 2007, 2011; Lewkowicz and Ghazanfar, 2009; Brandwein et al., 2011; Burr and Gori, 2012). While there is substantial work in animal models on the neural underpinnings of the development of MSI in single neurons, the majority focus on the emergence of these processes in anesthetized animals (Wallace and Stein, 1997; Wallace et al., 2004, 2006; Xu et al., 2012; Yu et al., 2013; Stein et al., 2014). Thus although great strides have been made in understanding the neural circuits necessary for the emergence of MSI and how this is impacted by environment (Wallace and Stein, 1997; Wallace et al., 2004, 2006; Cuppini et al., 2011b, 2012; Xu et al., 2012; Yu et al., 2013; Stein et al., 2014), the neural basis of the development of MSI for complex multisensory signals such as speech is not yet well understood. To make headway on this front, here we used a set of previously collected behavioral data (Foxe et al., 2015; Ross et al., 2015) to test a neuro-computational model of the development of multisensory speech perception.

A neural region of particular interest for the maturation of speech-related MSI is the posterior superior temporal gyrus/sulcus (pSTG/S), a cortical association area involved in speech perception (Rauschecker, 2011; Molholm et al., 2014) that is also implicated in audiovisual multisensory processing (Beauchamp et al., 2004; Saint-Amour et al., 2007; Matchin et al., 2014; Erickson et al., 2015). In addition, converging evidence reveals that MSI also occurs at very early stages of cortical processing of sensory inputs (Giard and Peronnet, 1999; Molholm et al., 2002; Foxe and Schroeder, 2005; Mercier et al., 2013, 2015), and it is highly likely that MSI occurs between auditory and visual unisensory regions prior to auditory and visual speech information converging on neurons within the multisensory processing hubs of the pSTG/S. As such the maturation of MSI in pSTG/S must be considered in the context of its feedforward inputs from auditory and visual cortices (Foxe and Schroeder, 2005; Schroeder and Foxe, 2005). Within the framework of this neural model, we hypothesized that the ability to benefit from multisensory speech results from a learning process during which speech representations informed by feedforward inputs from auditory and visual cortices are refined in pSTG/S. This model predicts that multisensory experience, not only improves multisensory perception, but also leads to comparably improved unisensory speech perception. This is due to the reinforcement of speech representations in pSTG/S in the case of feedforward projections, and/or of lower level speech representations in earlier auditory and visual association cortices in the case of feedback projections. Further, the reinforcement of direct cross-sensory connections (for discussion of such cortico-cortical connectivity see Meredith and Allman, 2009; Meredith et al., 2009) between auditory and visual speech representations in unisensory cortices might also play a role in the developmental trajectory of multisensory influences on speech perception.In this case, it can be assumed that synaptic connections among unisensory areas are initially relatively ineffective, but that they strengthen as a consequence of relevant multisensory experiences through a Hebbian learning mechanism.

The aim of the present work was to test a neural network model of multisensory speech perception informed by neurophysiology and its ability to explain behavioral speech recognition data. In particular, we wished to explore possible mechanisms underlying the maturation of multisensory integration by testing the model's ability to reproduce different empirical results reported in the literature concerning audiovisual speech perception, including the role of MSI in identification accuracy (Foxe et al., 2015) and its ability to produce the well-known audiovisual speech illusion, the McGurk effect (McGurk and MacDonald, 1976; Saint-Amour et al., 2007).

Moreover, there is compelling evidence that multisensory processing is substantially impaired in younger children with an autism spectrum disorder (ASD) (Foss-Feig et al., 2010; Kwakye et al., 2011; Brandwein et al., 2013, 2015; de Boer-Schellekens et al., 2013a; Stevenson et al., 2014a,b; Foxe et al., 2015), but also that these MSI deficits in ASD largely resolve during the adolescent years (de Boer-Schellekens et al., 2013b; Foxe et al., 2015). Importantly, multisensory processing deficits in ASD are likely to represent impairment of neural processes unique to MSI as it appears that they cannot be fully explained on the basis of unisensory deficits (Foxe et al., 2015). As of yet, the neural bases of this impairment remain unknown, and thus with the present model we also wished to provide possible explanations of the neural processing differences underlying the slower maturation of MSI in participants with ASD.

The model is based on a previous neural network implemented to study cortical multisensory interactions (Magosso et al., 2012; Cuppini et al., 2014) and consists of a multisensory region (assumed here to be pSTG/S) receiving excitatory projections from two arrays of unisensory neurons: the first (auditory region) devoted to the representation of auditorily communicated units of speech and the second (visual region) to the representation of visually communicated speech (i.e., lip and face movements; see e.g., Bernstein and Liebenthal, 2014). In the following, the network is first explained, including mechanisms underlying multisensory speech integration in pSTG/S neurons. Subsequently, we describe the training mechanisms implemented to simulate the maturation of speech perception. Parameters of the learning mechanisms are set to simulate the maturational trajectory in typically developing (TD) subjects from 5 to 17 years old, from behavioral data reported in the literature (Foxe et al., 2015). Finally, alternative hypotheses to characterize the different maturational trajectories in TD and ASD are critically discussed. In particular, three different conditions are tested to explain ASD deficits in speech MSI: reduced multisensory experiences during the maturation process due to altered attentional biases in children with autism (attentional bias), altered synaptic plasticity (learning bias), and decreased connectivity across the network (architectural bias). We will discuss the plausibility of these hypotheses as explanations for the delayed development of speech MSI in ASD, comparing simulated responses with behavioral data in ASD subjects (Foxe et al., 2015).

## Methods

In the following, the model structure and the training are described qualitatively. The mathematical description including all equations is provided in the Appendix (Supplementary Material), together with criteria for parameter assignment and parameter values [see Table 1 in Appendix (Supplementary Material)].

### Basal model: qualitative description

**Figure 1:**
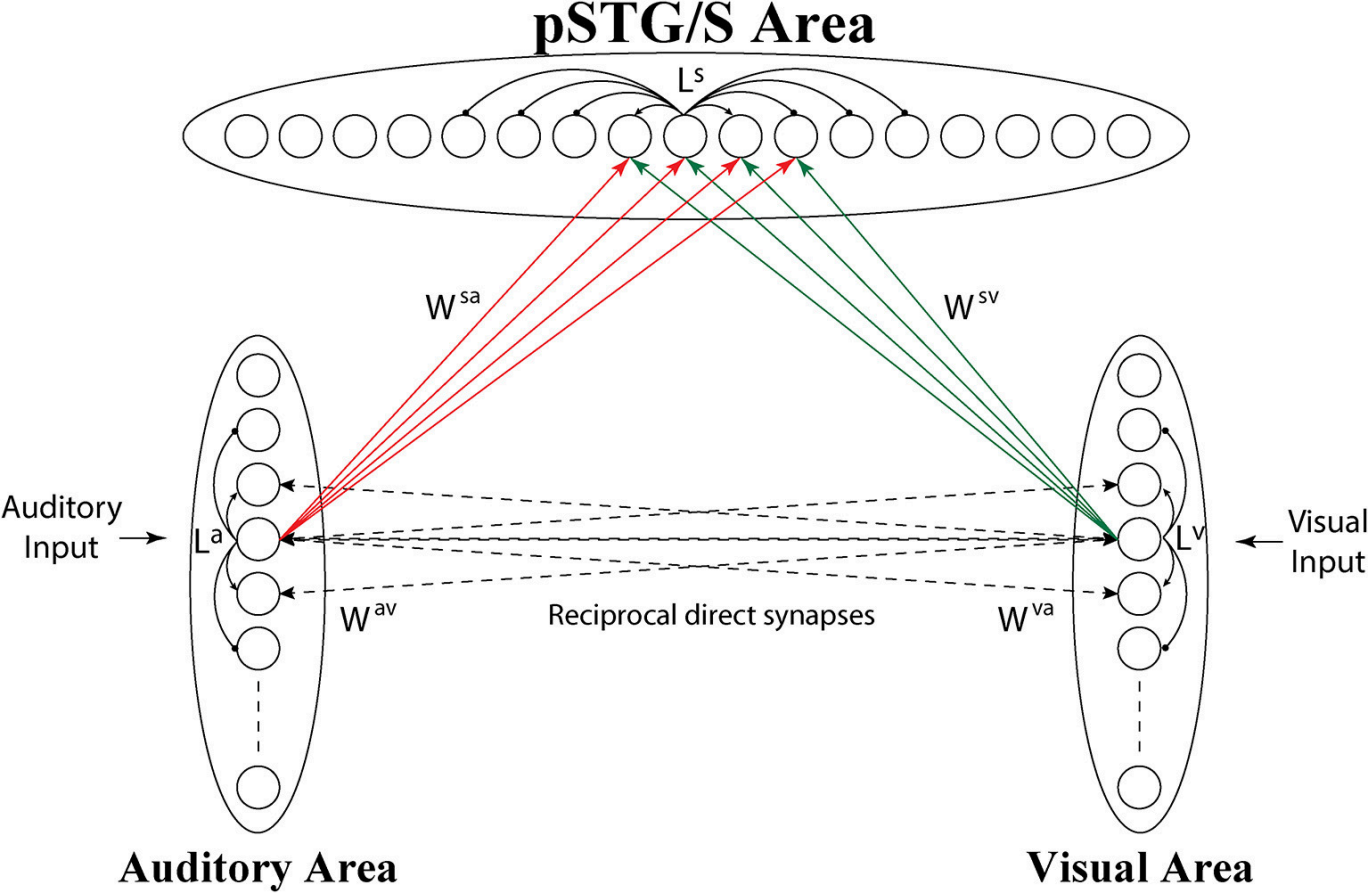
Architecture of the network. Each circle represents a neural population, i.e., a group of neurons coding for a given phoneme. Dashed lines represent connections (*W*^*av*^, *W*^*va*^) acquired during cross-sensory training, which simulates associative learning between speech sounds and gestures. Neurons belonging to the same region are also reciprocally connected through lateral connections (*L*^*a*^, *L*^*v*^, and *L*^*s*^, black solid lines), described by a Mexican Hat function (a central excitatory zone surrounded by an inhibitory annulus). Neurons in the unisensory regions send excitatory connections, reinforced during the training, (solid lines *W*^*sv*^, *W*^*sa*^) to the corresponding elements in the multisensory area.

The model consists of 3 arrays of N auditory, N visual and N multisensory neurons (the number of elements is arbitrary here, but was set at 100) (see Figure 1).

Neuronal responses to any input are described with a first order differential equation, which simulates the integrative properties of the cellular membrane, and a steady-state sigmoidal relationship that simulates the presence of a lower threshold and an upper saturation for neural activation. The saturation value is set at 1, i.e., all outputs are normalized to the maximum. In the following, the term “activity” is used to denote neuron output.

Each auditory and visual unit in the model is intended to represent a collection of neurons that when active together code for the speech sound and speech gesture of a given phoneme (for auditory and visual inputs, respectively). Elements in the unisensory regions are topologically organized according to a similarity principle. This means that two similar sounds or lip movements activate proximal neural groups in these areas.

The topological organization in these regions is realized assuming that each element is connected with other elements of the same area via lateral excitatory and inhibitory connections (intra-area connections, *L*^*a*^ and *L*^*v*^ in Figure 1), described by a Mexican hat distribution, i.e., proximal units excite reciprocally and inhibit more distal ones. This distribution produces an “activation bubble” in response to a specific auditory or visual input: not only the neural element representing that individual feature is activated, but also the proximal ones linked via sufficient lateral excitation. This arrangement can have important consequences for the correct perception of phonemes, for instance resulting in illusory perceptual phenomena like the McGurk effect (see section Results). In this work, it is assumed that topological maps are already largely developed through experience (Hertz et al., 1991), and lateral intra-area connections are not subject to training.

Furthermore, neurons in the auditory and visual regions also receive input (corresponding to a speech sound and/or a gesture representation of the presented phoneme). These visual and auditory inputs are described with a Gaussian function. The central point of the Gaussian function corresponds to a specific speech sound/gesture, and its amplitude with the stimulus intensity; the standard deviation accounts for the uncertainty of the stimulus representation. In this model, for simplicity, the two inputs are described with the same function. During the generation of words, speech gestures tend to onset prior to their accompanying acoustic energy and to be longer in overall duration; hence, each auditory stimulus lasts 80 ms, whereas each visual stimulus lasts 130 ms, and is presented to the network 50 ms before the auditory one. Onset and duration of these stimuli are chosen to mimic the experimental setup of Foxe et al. (2015). Moreover, to reproduce experimental variability, the external input is convolved with a noise component, taken from a uniform distribution.

Finally, we consider the existence of a cross-sensory interaction between the two unisensory areas. This cross-sensory input is computed assuming that neurons of the two areas are reciprocally connected via long-range excitatory connections (*W*^*av*^, *W*^*va*^), described by means of a weight factor, but also with the inclusion of a *pure latency*. The latter represents the time necessary for information to propagate from one neural unit to another along the connection fibers, i.e., the time during which the target neuron has not received the incoming input yet. We assume that, in the network's initial configuration, corresponding to an early period of development, cross-sensory connections have negligible strength.

The third downstream area simulates multisensory neurons in a cortical region (pSTG/S) known to be involved in speech processing and MSI (Beauchamp et al., 2004; Saint-Amour et al., 2007; Matchin et al., 2014; Erickson et al., 2015). These elements receive excitatory projections from units in the two unisensory layers, coding for auditory and visual representations of the same speech events, and are reciprocally connected via lateral connections with a Mexican-hat arrangement, implementing a similarity principle (*L*^*s*^).

Inputs to the multisensory area are generated by long-range excitatory connections from unisensory regions (*W*^*sv*^, *W*^*sa*^), and we use a delayed onset (pure latency) to mimic the temporal aspects of these inputs and a weight factor. We assume that in the initial configuration, the connections between unisensory and multisensory regions are symmetrical and characterized by poor efficacy. We chose this initial synaptic configuration to minimize the model assumptions and simulate an immature ability of the network to detect speech percepts, irrespective of sensory modality.

Finally, the output of the pSTG/S neurons is compared with a fixed threshold (30% of the maximum neurons' activity) and the barycenter of the suprathreshold activity in this layer is computed (subthreshold activity is just considered noise and is neglected in this computation), to mimic the perceptual ability to correctly identify speech (detection threshold). The recognized phoneme is the one closest to the barycenter. The network performs a correct recognition if this phoneme is equal to the one provided as input.

### Training the network

Starting from the initial immature configuration, we simulated the maturation of connections from a fully immature system to one that was at maturity [e.g., 0 years age to adulthood (17 years)]. To model typical experience with speech stimuli we made a simplified choice in which during the training period we stimulated the network with 65% of congruent cross-sensory auditory and visual stimuli and 35% of unisensory auditory stimuli. These values were not available in the literature, and presumably differ considerably across individuals and across the lifespan depending on circumstances. Unisensory visual cues were excluded from training since it is rare to encounter a person speaking without also hearing the corresponding phonemes. We chose the configuration that best replicated the behavioral results for TD subjects in Foxe et al. (2015). Extensive simulations using different proportions showed that multisensory learning increased as a function of multisensory experience. Training involved 8,500 exposures, at which point the network produced mature-like behavior. Stimuli were generated through a uniform distribution of probability. We used stimuli at their highest level of efficacy, i.e., able to excite unisensory neurons close to saturation, in order to speed up the modeling process. During this period, both the feedforward connections to the pSTG/S area and the direct excitatory connections between the unisensory regions were modified by using a simple rule for connection learning (consisting of Hebbian reinforcement and a decay term). As specified above, intra-area lateral connections were not subject to training. In particular, the training algorithm reinforced the connections on the basis of the correlation between the activities in the pre-synaptic and post-synaptic neurons (Hebb rule). The decay term was proportional to the activity of the post-synaptic neuron, and included a scaling factor that established the maximum saturation value for the connection (see Appendix for more details).

The parameters of the synaptic learning rules (the learning factors, and the upper saturation for the synaptic weight) were assigned to simulate the data by Foxe et al. (2015) concerning maturation in the TD group. Moreover, to attain satisfactory reproduction of the experimental data, we used different values for these parameters in the feedforward connections (*W*^*sa*^ and *W*^*sv*^) compared with the cross-sensory connections (*W*^*av*^ and *W*^*va*^). This reflects that cross-sensory inputs to unisensory areas have been shown to elicit modulatory responses, whereas, as far as we know, they have not been shown to elicit action potentials (Allman and Meredith, 2007; Allman et al., 2008; Meredith and Allman, 2015). It is worth noting, however, that we used the same parameters in the auditory and visual branches of the network; therefore differences in network abilities in cases of auditory and visual stimulation emerge as a consequence of differences in the sensory experience with speech stimuli during the training phase (not in the parameters).

Finally, in order to compare our results with those of Foxe et al. (2015), we needed to relate the number of epochs during training with the subject's age. This choice, of course, depended on the values used for the learning rate (the higher the learning rate, the smaller the number of epochs). In the network's initial state, there was no speech-detection ability. With the parameters used, training with 2,500 epochs led to an architecture configuration yielding unisensory and multisensory performance comparable to the 5-year-old subjects (the first data point present) in Foxe and colleagues' data (Foxe et al., 2015). Therefore, in what is undoubtedly an oversimplification, 500 epochs of training were assumed to correspond to 1 year of experience. According to this linear approach, 8500 epochs corresponded to exposures of a 17 year old, the oldest age represented in Foxe et al. (2015).

After training, the model behavior was assessed as described in the next sub-section. Finally, we implemented and tested different structural or functional assumptions to simulate the delayed development of speech MSI in ASD children. Specifically, we trained the network assuming: (1) reduced attention/exposure to visual articulatory information (due to, for example, reduced fixations to the face due to face avoidance perhaps, intact fixation but reduced attention, both, and so forth), simulated by eliminating ¼ of visual inputs in case of multisensory experience (hence, cross-sensory inputs become merely auditory in ¼ of cases). We assumed that attentional biases are partly and progressively overcome by interventions and/or naturally occurring developmental changes. In accordance with this notion, the number of multisensory events was progressively increased with age, to reach a TD-like multisensory experience in the final stage of the development. (2) A different level of synaptic plasticity, with the learning rate set 2 times lower for modeling of the ASD behavior. (3) Reduced interregional connectivity, with fewer connections among all regions of the model (i.e., 10% of connections are lacking).

### Assessment of network performance

We performed several simulations to test network behavior before, during, and at the end of the training process, modeling performance for unisensory (auditory-alone and visual-alone) and multisensory inputs (congruent and incongruent audiovisual representations).

#### Speech recognition task

The network was stimulated with inputs simulating an auditory-alone, a visual-alone, or a congruent visual-auditory (multisensory) speech event. As described above, the speech event was assumed to be correctly recognized if the barycenter of the evoked activity above the detection threshold (30% of the maximum activity) in the pSTG/S area matched the element coding for the speech event presented as input to the model. We used different levels of auditory input amplitude, ranging from ineffective (which minimally activated the auditory speech region and generated 0% correct identifications in the model, efficacy level of 1) to a maximum level (able to saturate the auditory evoked activity and generated more than 80% correct phoneme identification in the adult, efficacy level of 7). The use of different auditory efficacy levels allowed us to mimic speech recognition at different auditory signal to noise ratios, as in previous work (Ross et al., 2011; Foxe et al., 2015). In contrast, the efficacy of the visual stimulus was held constant: we chose a visual level so that, in the adult configuration, the model presented a poor ability to detect speech based on visual information alone. Critically, this mimicked what we see in our experimental work (Foxe et al., 2015).

Since the presence of noise introduced variability in the network's outcome, for every level of efficacy of auditory input, unisensory and multisensory speech-detection abilities were evaluated for 100 speech events. To evaluate the acquisition of speech perception under unisensory and multisensory conditions, we computed the mean responses across all levels of input efficacy at different epochs of training.

#### Speech recognition time

The network was stimulated with an auditory or a congruent visual-auditory (multisensory) speech representation and we evaluated the time necessary for the pSTG/S neuron coding for the specified speech unit to reach the detection threshold (30% of its maximum activity). The configuration of the inputs and the simulations were the same as in the previous task. The mean response (in terms of recognition time) over all the 100 outcomes at each level of efficacy was computed separately for the unisensory and multisensory conditions. Moreover, we evaluated these data both at an early stage of maturation (after 2,500 training epochs), and in the mature configuration.

#### McGurk effect simulations

We assessed whether the network was able to reproduce the McGurk effect, whereby conflicting auditory and visual speech inputs lead to an illusory speech percept that did not correspond to the percept evoked by the same auditory input when presented in isolation. In this case, the network was stimulated with mismatched visual-auditory speech inputs, with the visual representation shifted by 4 positions (i.e., outside the receptive field of the veridical corresponding speech unit) with respect to the auditory one. During these simulations: (i) we verified whether the activity in the multisensory area overcame the detection threshold; (ii) in case of detection, phoneme recognition was assessed by computing the barycenter of the supra-threshold activity in the multisensory region, and approximating the closest phoneme. We assumed that the McGurk effect occurred when the detected phoneme was different from that used in the auditory input. Each phoneme was stimulated 20 times by its auditory representation at each level of efficacy, coupled with a visual representation of a 4 position-distance phoneme, and the network response was averaged over all phoneme representations and all levels of auditory input efficacy. We also assessed whether the network was sensitive to the McGurk effect at different training epochs.

## Results

In the following we critically analyze the network behavior at different stages of training to highlight the developmental trajectory of MSI until the model reaches its final adult-like synaptic configuration. To this end, the network was repeatedly stimulated with auditory, visual or visual-auditory representations of speech events, at different levels of efficacy (corresponding to different SNR levels). Mean response in terms of correct speech recognition in cases of auditory presentations are compared with the correct speech identifications in cases of multisensory stimuli for all the different training epochs analyzed to compute the acquired MSI abilities of the network. Finally, additional multisensory tasks were simulated (see methods for details) to better characterize the acquisition of integrative abilities by the network.

### Modeling the development of audiovisual speech perception: training the network

**Figure 2:**
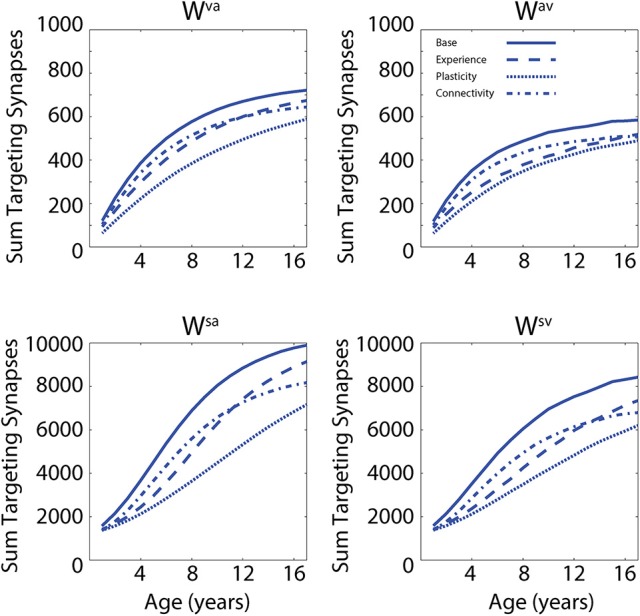
Synaptic maturation vs. multisensory experience. Examples of the reinforcement of cross-sensory connections **(upper)** and of feedforward connections **(bottom)** during the training phase in case of TD training (solid lines, AV 65%, A 35%) and trainings with (1) reduced multisensory experience (dashed lines, AV 40%, A 60% in the initial stage of training phase); (2) reduced synaptic plasticity (dotted lines, 50% of the learning rate used in the TD training); and (3) reduced connectivity (with 90% of intact synapses, dash-dotted lines). In panels, x-axis reports the simulated age (1 simulated year corresponds to 500 training epochs) and y-axis reports the sum of all the excitatory connections targeting all elements in the post-synaptic region. In the left upper panel, we report the sum of connections, *W*^*va*^, from all auditory elements targeting the visual area; the right panel represents the sum of all the connections, *W*^*av*^, targeting the auditory area, from all visual elements. The two bottom panels show the sum of the connections targeting the multisensory area from the elements in auditory region (connections *W*^*sa*^, left) and from the elements in the visual area, (connections *W*^*sv*^, right).

In a first set of simulations, we analyzed the modifications of the network's architecture as a consequence of the training conditions. In addition, the effects of different “perturbations” (either in terms of sensory experience, network parameters, or network architecture) to the network on the developmental trajectories of unisensory and audiovisual speech perception are considered in order to test possible explanations of the delayed development of speech MSI that is seen in ASD (Foxe et al., 2015). In Figure 2, we present the maturation of the networks excitatory projections under the four different conditions: in the case of so-called typical development (solid lines); training with lower efficacy multisensory experiences (dashed lines); training with reduced plasticity of the network's connections (dotted lines); and training of the network under conditions of reduced connectivity (dash-dotted lines). These four trainings produced substantially different patterns of maturation of the excitatory projections. The upper panels report the sum of all the connections targeting the visual region, coming from the auditory region (*W*^*va*^, left panel) and the sum of all the connections targeting the auditory elements, coming from the visual region (*W*^*av*^, right panel). The lower panels report the sum of the feedforward projections (*W*^*sa*^ and *W*^*sv*^) targeting the multisensory elements, from the two unisensory regions.

In the first case, by using a configuration of parameters and training experience that mimicked typical development (solid lines, audio-visual stimuli 65% and auditory-alone stimuli 35%), the network presented a quick increase of the feedforward and the cross-sensory connections. It is worth noting that due to the chosen training rule and the kind of experience used during this phase, the feedforward and cross-sensory connections coming from the auditory region are always stronger than the corresponding connections coming from the visual region, and this persists throughout maturation, much as one would expect. Reduced multisensory experience (dashed lines, 25% less of audio-visual stimuli at the beginning of the training period) produced a slower maturation of the connections in the network, which led to a weaker synaptic configuration for both the feedforward and the cross-sensory connections, as compared with the resulting connectivity after the basal multisensory training. In the case of reduced plasticity (50% of the learning rate used in the TD training), the final configuration of the network presented very poor synaptic efficacy. Finally, in the case of limited connectivity (90% of intact synapses, dash-dotted lines), the maturation of network's connections followed a profile similar to the case of reduced multisensory experience over the initial training epochs, but the feedforward synapses, especially from the auditory area, were found to be less effective in the final configuration of the model (adult stage).

### Testing network behavior against empirical data

**Figure 3:**
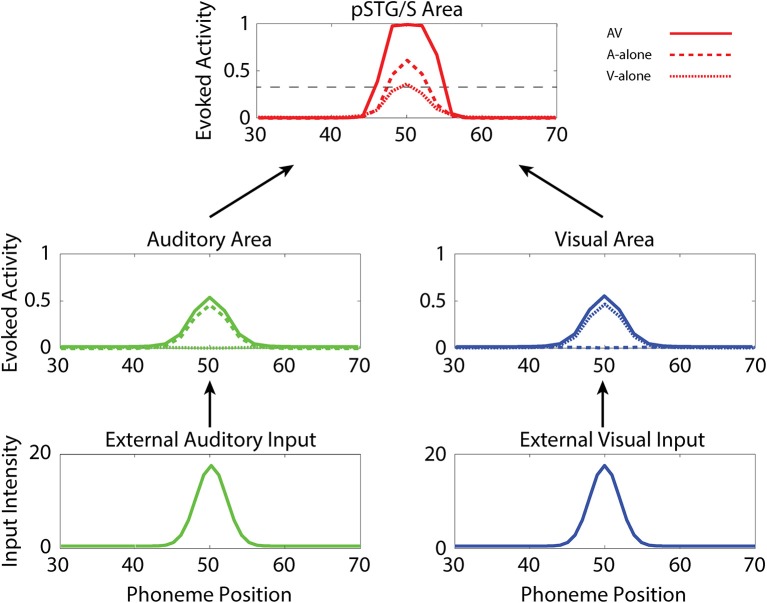
Auditory-alone, visual-alone, and audio-visual stimulation. Three exempla of network activity, in the different layers, in response to unisensory (auditory or visual) or cross-modal stimulation. When stimulated by an auditory or a visual unisensory input (dashed and dotted lines, respectively), the network in its mature configuration exhibits a low activity in the multisensory area. This is a condition of difficult speech-detection. Depending on the noisy component added to the external input, the overall activity in the multisensory area may remain below the detection-threshold (fixed at the 30% of its maximum value, black dotted line), or moderately rise above it. Conversely, when the network receives a multisensory input (solid lines), the activity in the two modality-specific regions exhibits a further increase (due to cross-sensory connections) and the evoked activity in the multisensory area reaches the maximum value, well above the detection threshold (multisensory enhancement), simulating a condition of more likely speech-recognition.

In the following, we first analyze the abilities acquired after training under “typical” conditions. Then we compare these results with those of the same simulations but with (1) reduced multisensory exposures, (2) lower synaptic plasticity, and (3) lower connectivity. Figure 3 illustrates the behavior of the network in the cases of unisensory (auditory, dashed lines, and visual, dotted lines) and multisensory (solid lines) simulations. Each panel describes the activities of the central neurons in each area (between positions 30 and 70) at maturity. External sensory inputs, described through a Gaussian function, elicit the activation of unisensory representations in the corresponding areas, which in turn excite the multisensory elements through the feedforward projections. The barycenter of the supra-threshold evoked activity in this region determines the phoneme identified by the network. This particular simulation refers to the mature network, i.e., after 8,500 training epochs, under basal (TD-simulating) conditions. Worth noting is the strong enhancement in the pSTG/S area in case of multisensory inputs.

### Developmental process and audio-visual speech recognition

**Figure 4:**
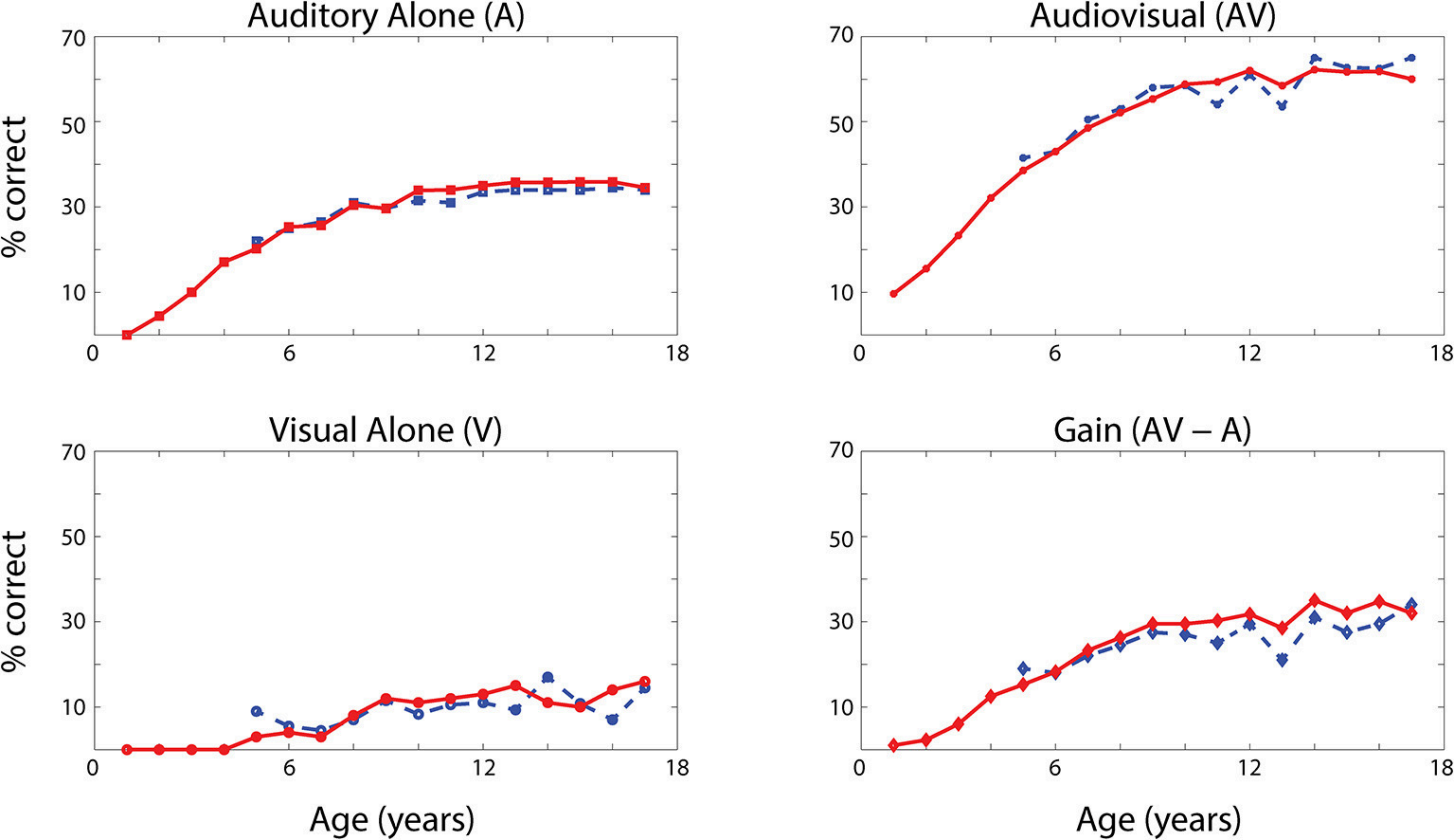
Speech recognition performance in the auditory-alone (A), visual-alone (V), and audiovisual (AV) conditions, and AV gain (AV–V), as a function of training epochs. Average speech recognition performance of the model (% correct) evaluated at different epochs during the training (red solid lines). The network results are compared with experimental data from Foxe et al. (2015) where the speech-recognition tasks were evaluated at different ages (5–17 years) during the adolescence (blue dashed lines). The upper and lower left panels report the percentage of correct speech recognition (y-axis) when the network was stimulated with the auditory input only (A) and visual input only (V), at different epochs of maturation. Upper right panel shows results of simulations with congruent visual and auditory representations simultaneously presented to the network [multisensory stimulation (AV)]. Finally, lower right panel depicts the maturation of the multisensory gain, computed as the difference between the percentage of correct detections in case of multisensory stimulations and its counterpart in case of auditory-alone stimuli (AV-A). These data represent the mean of correct recognitions over 700 different presentations at each training stage.

The network's ability to correctly identify speech events was evaluated at different levels of efficacy and different phases of maturation. Figure 4 describes the network behavior, in terms of correct speech detection (red solid lines), under conditions of unisensory (auditory, A, and visual, V) and congruent auditory-visual (AV) stimulation. Moreover, the lower-right panel shows the multisensory gain, computed as the difference between auditory-visual and auditory-alone performance (AV-A). For every condition, the model behavior was averaged over all speech representations and all levels of auditory input efficacy to assess the performance of the model. This was done at different phases of training, simulating years of age (from no training to near asymptotic model performance, i.e., adult condition).

The output of the network was compared with the acquisition of audio and audiovisual speech-recognition capabilities as described by Foxe et al. (2015). The agreement is good, as expected, since the parameters of the model were set to reproduce these data. In both cases (see Figure 4, behavioral data, dashed lines, and simulation results, solid lines), speech-perception exhibited similar maturational trajectories, both in the case of visual-alone and auditory-alone stimulation as well as in the enhanced speech recognition for multisensory inputs. The network reached “adult-like” abilities in the case of unisensory stimulations after 5,000 training epochs (approximate corresponding age: 10 years), and multisensory speech-detection abilities after 6,500 epochs (simulating 13 years of age). Under unisensory conditions, the capacity to correctly perceive speech from visual gestures remains much smaller than the capacity to perceive words from auditory information, even in the mature stage of the network.

It is worth noting that the maturation of unisensory abilities does not fully mirror the developmental trajectory of multisensory speech abilities. Indeed, the acquisition of the former reflects only the reinforcement of the within-modal feedforward connections (i.e., the feedforward auditory, W^sa^, in the case of phoneme detection, and the feedforward visual, *W*^*sv*^, in the case of gesture detection). Conversely, the development of multisensory speech recognition reflects two simultaneous mechanisms: (i) the presence of cross-sensory connections (*W*^*av*^ and *W*^*va*^) among elements in the unisensory areas, which potentiate the unisensory activities; (ii) the enhancement in the multisensory region, due to the simultaneous cross-convergence of auditory and visual feedforward excitation to the same neuron, causing multisensory enhancement. In particular, as shown in Figure 3, even moderate activities in unisensory regions can evoke strong multisensory activity if they occur in temporal proximity.

**Figure 5:**
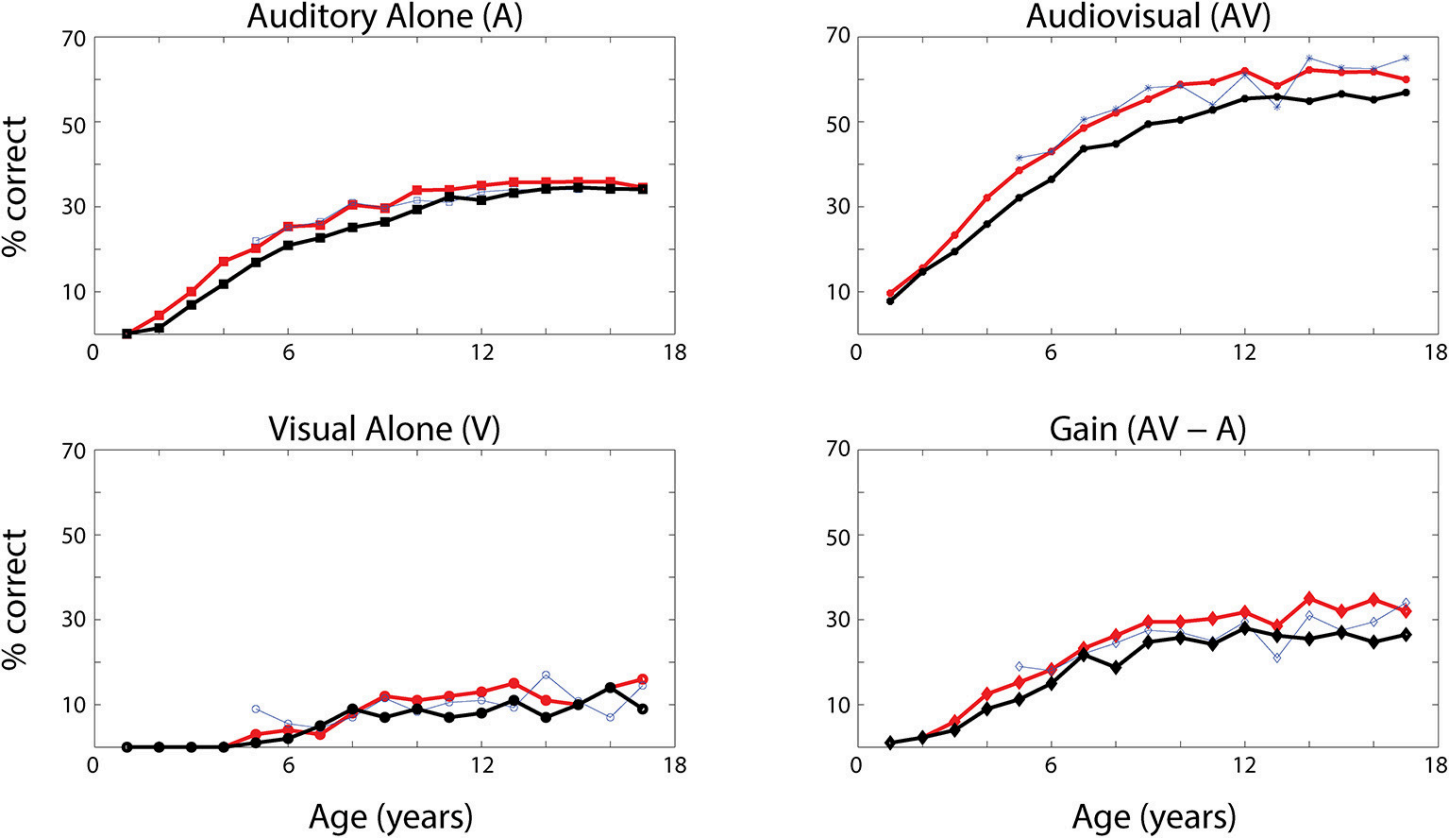
Speech recognition performance with ineffective cross-sensory connections. Average word recognition performance of the model (% correct) evaluated at different phases during the training (solid lines) in case of A-alone, V-alone and AV stimuli. The red lines represent the same results as in Figure 4, with all connections intact. The black lines have been obtained assuming no reinforcement of cross-sensory connections (γ^*c*^ = 0 and so *W*^*av*^ = *W*^*va*^ = 0). The results are compared with experimental data from Foxe et al. (2015). These data show that the cross-sensory connections have a negligible effect in the unisensory conditions, but they affect the multisensory abilities of the network. As in Figure 4, these data represent the mean of correct recognitions over 700 different presentations at each training stage.

To test the role of cross-sensory connections, we repeated the previous simulations without cross-sensory links, (i.e., we set *W*^*av*^ = *W*^*va*^ = 0). The results are reported in Figure 5 (red lines, simulations with the intact network, black lines, simulations with ineffective cross-sensory connections). While the speech-perception abilities in case of unisensory stimuli are almost the same, multisensory performance is reduced by the impaired cross-sensory connections.

### Simulation of multisensory facilitation of speech recognition times

**Figure 6:**
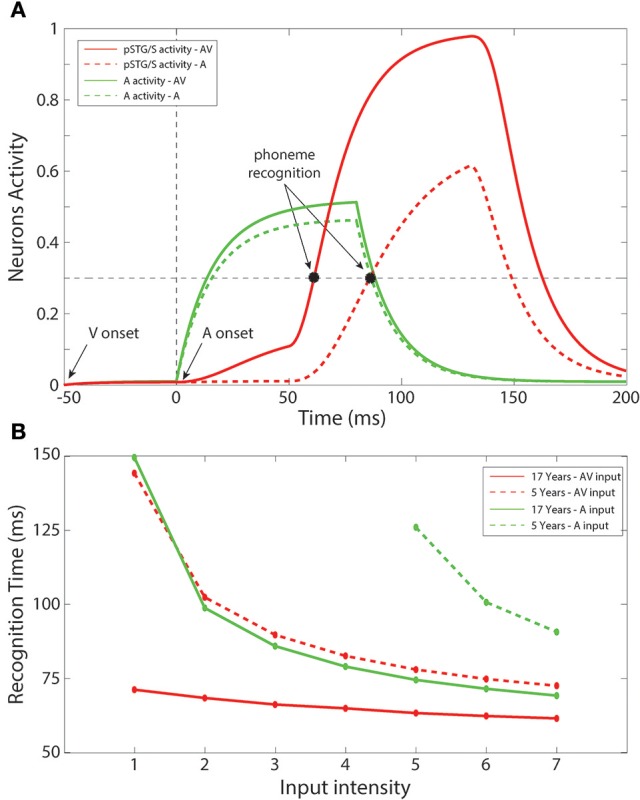
Recognition times. **(A)** Auditory-alone vs. multisensory simulations. Activities in the auditory (green lines) and in the multisensory (red lines) areas of the model, evoked in case of auditory-alone (dashed lines) and visual-auditory (solid line) stimulus, and compared with the detection threshold (equal to the 30% of the maximum evoked activity). The unisensory configuration is able to generate a phoneme recognition at about 85 ms after the auditory presentation, while the multisensory stimulation leads to a correct phoneme recognition 21 ms earlier than the unisensory case. It is worth noting the effect of the visual component on the auditory neurons through the mature cross-sensory connections *W*^*av*^: in case of an AV stimulation the model presents a quicker and stronger activity in the auditory area. The quicker activation of the multisensory region depends both on the quick auditory activity and, above all, to multisensory enhancement. **(B)** Unisensory vs. multisensory condition, at 5 years old and in the adult configuration. Mean times for a correct speech recognition have been computed at different input levels in case of stimulations with auditory representations only (green lines), and with auditory and visual representations simultaneously (red lines), in an early training stage, 5 years of age, (dashed lines) and in the adult phase (solid lines). The multisensory configuration produces faster responses also in the immature stage. Moreover, with the adult network architecture, a multisensory presentation leads to speech recognition 12–70 ms earlier than a corresponding auditory stimulation, in agreement with results by Arnal et al. (2009).

In a subsequent series of simulations, we simulated recognition times for speech under unisensory (auditory) vs. multisensory conditions. Figure 6A shows an example of the network temporal response under multisensory vs. unisensory stimulation. The figure displays the evoked activities in the multisensory region (red lines) and in the auditory area (green lines) in response to a sample auditory stimulus of middle efficacy presented alone (dashed line) or coupled with a visual stimulus (solid line). In the multisensory case, the visual input sent an additional excitatory component to the auditory units through the mature connections among elements of the unisensory regions; this led to quicker activation of the auditory area. Activation of the pSTG/S area, under multisensory conditions, is even quicker due to two combined phenomena: the quicker response in the auditory area, and the convergence of two feedforward inputs to the same multisensory region, resulting in strong enhancement. The overall effect of these mature excitatory connections was thus to speed up the activation of pSTG/S in response to audiovisual speech, and correspondingly to reduce the speech recognition time. In particular, in response to multisensory stimulation, the network recognized the speech input after just 64 ms; while the presentation of the auditory-alone speech inputs led to recognition times that were delayed by 21 ms (reaction-times presented here should be considered relative to each other rather than veridical). Correct recognition time was defined as the time when the activity elicited in a neuron in pSTG/S region overcomes the “detection threshold,” fixed at 30% of its maximum value.

Figure 6B summarizes mean speech recognition time computed for each level of stimulus efficacy. A number of notable observations emerge. First, the simulations showed faster reaction times under less noisy/more effective stimulation conditions, and this was the case for all conditions. This phenomenon was observed both with the unisensory auditory and multisensory inputs, as well as in immature (5 years) and in mature (17 years) configurations. Second, the simulations showed multisensory facilitation of reaction times even in the immature network, but recognition times were faster for the mature configuration.

As in our earlier simulations, in the case of auditory-alone inputs, network performance was poor when stimulus efficacy (i.e., intensity or signal to noise ratio) was low; thus, there were no data-points for this condition for the 4 lowest input efficacy levels in the immature condition. For all other efficacy levels and conditions, recognition times ranged from 60 to 150 ms (This should not be thought of as veridical reaction time since it would be very rapid). For efficacy level 5, for which there were recognition times for all conditions considered, mean time of recognition ranged between 61 ms for the mature network under conditions of multisensory speech and 128 ms for auditory-alone speech in the immature stage of the network. As illustrated in Figure 6B, the benefit of audiovisual multisensory stimulation, if compared with the unisensory input, was as large as 12–80 ms for the mature network, and 20–50 ms in the immature condition with the larger improvement in cases of stimuli at the lower levels of intensity. If one were to extrapolate from neural facilitation to behavioral facilitation, these values are in general agreement with experimental data on multisensory based facilitation of neural responses, in speech perception tasks, from Besle et al. (2008) and Arnal et al. (2009). Moreover, the model RTs are in agreement with behavioral data reported in Besle et al. (2004), showing faster responses in cases of audiovisual speech stimulation.

### Effects of different “perturbations” to the network on the development of MSI

**Figure 7:**
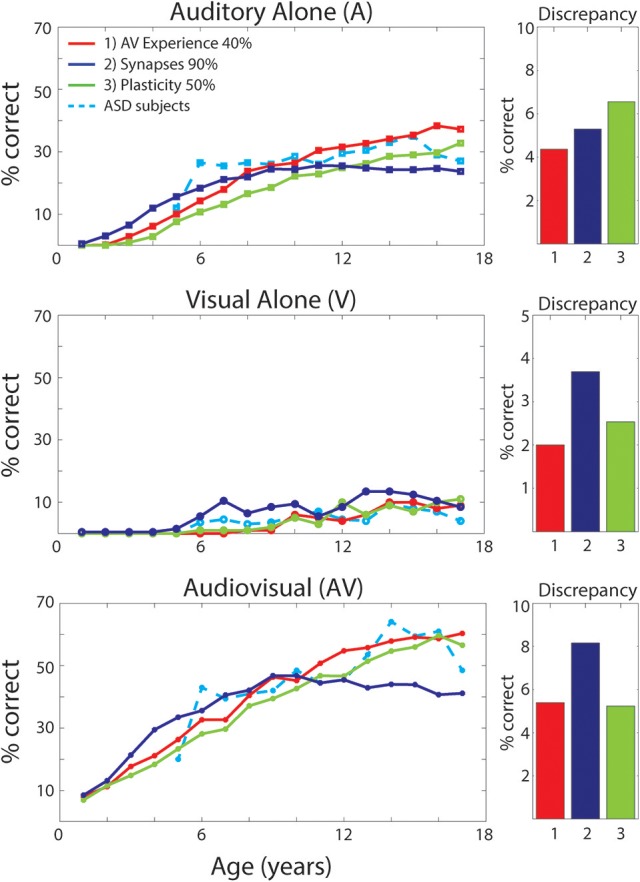
Speech recognition performance in the auditory-alone (A), visual-alone (V), and audiovisual (AV) conditions, in case of different “perturbations” to the network. Average speech recognition performances (% correct) evaluated at different epochs during “perturbed” synaptic maturation with: (1) reduced multisensory experience (40% AV in initial training condition—red solid lines); (2) reduced synaptic plasticity (50% learning rate—green solid lines); (3) reduced number of synaptic connections (90% of synapses—blue solid lines). The network results are compared with experimental data on ASD children during adolescence, as reported in Foxe et al. (2015) (light-blue dashed lines). The meaning of all panels is the same as in Figure 4. Histograms on the right report the discrepancy between model predictions and the experimental data on ASD children. The discrepancy is evaluated as the mean absolute error between simulated points and experimental points, for each perturbed training and each stimulation condition (A, V, AV).

As discussed previously (see Figure 2), different perturbations to the network will affect the maturation of the model's architecture differently. How each of these structural modifications translates into different model's behaviors in terms of speech-recognition abilities is displayed in Figure 7. The figure reports results of the simulated speech-recognition task, for each impaired training condition, at different training epochs, compared with behavioral results in ASD children described by Foxe et al. (2015) (light-blue dashed lines). Moreover, for each type of perturbation and each input configuration (A-alone, V-alone, congruent AV), Figure 7 displays the discrepancy between the model's behavior and the behavioral data, evaluated as the average absolute error between the simulation's results and experimental data reported by Foxe et al. (2015), for each age-group.

In the following, we critically analyse each training condition and the corresponding results, in order to identify which perturbation better explains the behavioral data of ASD subjects and their delayed MSI maturation.

#### Reduced multisensory exposures

Multisensory experience during the developmental period is fundamental for the acquisition of multisensory integrative abilities (Stein et al., 1999, 2014; Bahrick and Lickliter, 2000; Pons et al., 2009; Lewkowicz, 2014; Rowland et al., 2014; Xu et al., 2015). The same is correct also for this network. Not reported here for briefness, extensive simulations using different proportions between multisensory and unisensory stimuli showed that multisensory learning increased as a function of multisensory experience. Hence, we can suppose that one possible explanation for reduced MSI in ASD, is that these individuals experience fewer multisensory exposures, possibly due to how attention is allocated (e.g., suppression of unattended signals; selectively focusing on one sensory modality at a time; not looking at faces consistently). We therefore tested the impact of percentage of multisensory vs. unisensory exposures on the maturation of MSI. It is worth noting that, in these simulations, we just modified the percentage of cross-modal inputs, without any additional parameter change compared with the TD case. Specifically, we trained the network starting with 40% of AV stimuli and 60% A, and then we increased the multisensory experience by 1.5% every 500 epochs (simulating 1 year). In this condition, the network received a TD-like multisensory experience only at the end of the training period.

As already shown in Figure 2, reduced multisensory exposures led to weaker connectivity among the regions of the network. The speech-recognition abilities acquired by the model as result of such training are displayed in Figure 7 (red solid lines). It is worth noting that the maturation of the network's ability to detect phonemes follows a similar profile as the ASD subjects, for every stimulus condition. Moreover, comparing these results, obtained with a lower multisensory experience, with the simulated TD condition reveals that: in case of auditory inputs, after a slower development in the first few years, both conditions follow a similar profile and reached mature levels of behavior (see Figure 4) at the same age (after 10-years of age). Conversely, in the ASD condition the network displayed a slower acquisition of the visual ability to detect speech gestures, and a delayed maturation of MSI capabilities. However, although the ability to detect speech from visual inputs remains lower at the end of training (8,500 training epochs) compared to the basal condition, the network was able to reach almost the same TD-like behavior in terms of benefiting from MSI speech.

#### Reduced synaptic plasticity

Another explanation for differences in MSI in ASD is less effective learning mechanisms. To test this possibility, we explored the effects of reduced plasticity on the model and determined how good a fit to the actual data this provided, and how it compared with an attentional account as described above. It is worth noting that for these simulations, we only modified the learning rate of every trained connection of the network, while the percentage of cross-modal inputs and any additional parameter value did not change compared with the basal case.

Results reported in Figure 7 (green solid lines) of the simulated speech-recognition task display that *reduced plasticity* (50% than that used in the simulated TD condition) affects the maturation of both unisensory and multisensory abilities in a similar way. That is, both unisensory and multisensory performance is considerably impaired compared to the basal condition (Figure 4). In this condition, all maturation capacities (either unisensory or multisensory) became comparable to the TD condition only at the very end of the training period (8,500 training epochs). Conversely, in the behavioral results, auditory capacity develops more rapidly than the multisensory one.

To explore the effect of this parameter, we also performed a simulated development by using a plasticity equal to 70% of the basal value. Results are not displayed for briefness, but they support the previous finding: unisensory and multisensory abilities are equally impacted by this modification. As such, this does not provide a good account of the pattern of deficits seen in ASD, which are considerably greater for MSI.

#### Impaired integrity of excitatory projections between regions

To test the idea that impaired structural connectivity may lead to atypical multisensory speech performance in children with ASD, the model was trained with a structural bias such that 10% of the cross-modal and feedforward excitatory projections could not be strengthened. These, chosen randomly, were maintained always at zero. This connectivity pattern resulted in behavioral deficiency that could not be overcome even at the end of the training. Unisensory and multisensory performance of the model (solid blue lines in Figure 7) is considerably impaired compared to the basal condition and differs significantly from ASD behavioral data, as shown in Figure 7, even in its final configuration (after 8,500 training epochs).

As done in the previous case, also for this parameter, we ran a number of simulated developments with different degrees of structural impairment (30% and 50% impaired connections). Results are not shown for briefness, but supported our findings: the greater the impairment, the worse the unisensory and multisensory speech performance of the network.

### Simulation of the McGurk effect in “typical” development and ASD development

An important consequence of training in our model is that the audio-visual interference becomes stronger as training proceeds, because of connection reinforcement (Figure 4). This change should have important implications for the development of audio-visual speech illusions, the best-known of which is perhaps the McGurk illusion (McGurk and MacDonald, 1976; Saint-Amour et al., 2007). Moreover, since the network predicts different developmental trajectories for the synapses, it might provide different predictions as to the occurrence of the McGurk effect in the case of “typical” development and perturbed developments. In the network, the McGurk effect is evaluated by computing the network response to mismatched (at four-position distance) auditory-visual speech inputs. In our simulation, an outcome is considered a McGurk effect when the detected phoneme computed as the barycenter of activity in the multisensory regions is different from that used in the auditory input (see section Methods for details).

**Figure 8:**
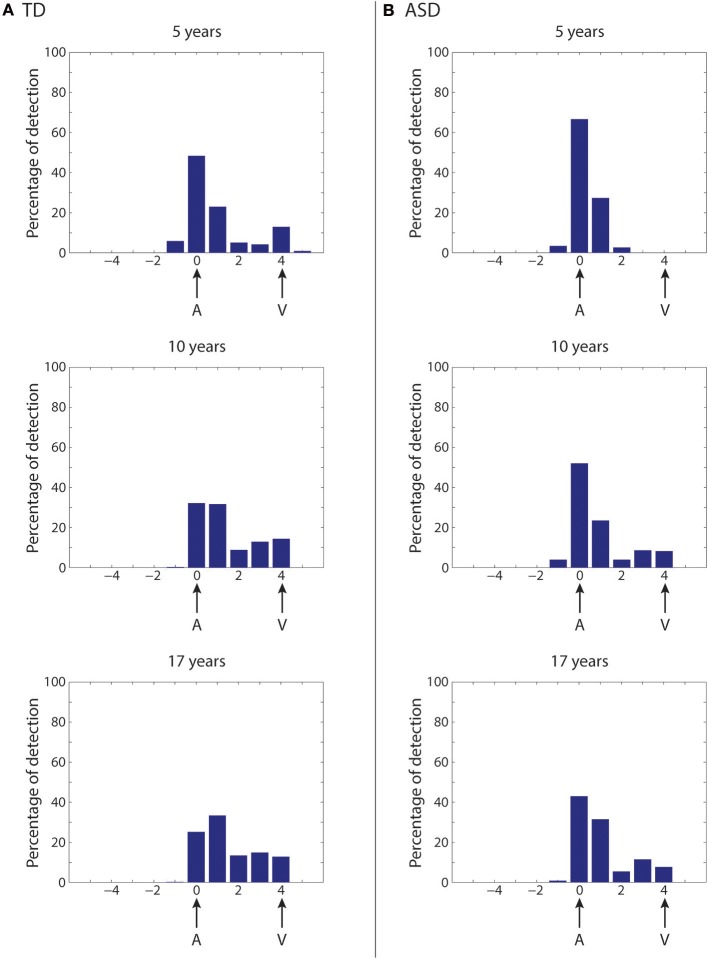
Simulation of the McGurk effect in typical development and in case of poor multisensory experience. Network response to McGurk-type situation, evaluated at different epochs of the network training in case of typical development **(A)** and in case of development under poor multisensory experience **(B)**. In both cases, mismatched (at four-position distance) auditory-visual speech inputs are used to test the network abilities. We define that the McGurk effect is evident when the detected phoneme (computed as the barycenter of activity in the multisensory region) is different from that used in the auditory input. Histograms (from top to bottom) show the percentage of auditory classifications for the network (see Methods for details) averaged over all phoneme representations and all auditory stimulus efficacies, in an early stage of training (5 years, upper panel), in an intermediate stage (10 years, middle panel), and in the adult (17 years) configuration (lower panel). X-axis shows the relative positions of the detected phonemes with respect to the position of the auditory representation. **(A)** (Typical development)—After 5,000 training epochs (10 years of simulated age) the model already shows a strong McGurk effect (percentage of correct phoneme detection as low as 33%), although this is not as strong as in its final configuration (simulations after 8,500 epochs of training, corresponding to 17 years). In this last configuration, the model presents the McGurk effect in almost 75% of cases. It is worth noting that when the network fails to recognize the correct phoneme, it identifies different phonemes that are a fusion of the visual and auditory inputs, but only rarely identifies the visual one. **(B)** (Development under poor multisensory experience)—The network results less susceptible to the McGurk illusion than the simulated TD condition. The network, even after 5,000 training epochs (corresponding to 10 years, Middle histogram), is characterized by a poor visual influence on the auditory percept; the correct auditory phoneme is still recognized in more than 55% of the cases. Only at the end of its maturation (Lower histogram), the network presents a greater McGurk effect, even if it is not yet comparable with that in the simulated TD condition (correct auditory phoneme recognition in 45% of the simulations).

First, we simulate the McGurk effect under “typical” developmental conditions (Figure 8A). The network trained with a rich multisensory experience quickly shows the influence of the visual modality on speech perception. After 2,500 training epochs (5-years of age), percent correct phoneme identification is about 50%. However, by 5,000 training epochs (10-years of age), when the network reaches “adult-like” abilities for auditory-alone and visual-alone stimulations, the McGurk effect is already clearly evident (percent correct phoneme identification at about 33%), although not as strong as in its final configuration. After 8,500 epochs of training (17-years), percent correct phoneme identification is about 25%. In this last state, in more than the 60% of the cases, the speech percept is a fusion of the two stimuli, and in about 15% of cases, the network identifies the visual phoneme.

As described in the previous section, in our network, reducing multisensory exposures leads to better agreement with the ASD data than the other two perturbations (Figure 7), providing support for an attentional account of impaired MSI in ASD. We follow this up by testing how well fewer multisensory exposures would impact the McGurk illusion compared to “typical development,” and whether network performance would align with the finding that children with ASD are less vulnerable to this illusion (Taylor et al., 2010; Irwin et al., 2011; Bebko et al., 2014; Stevenson et al., 2014b). To this aim, the same simulations as in Figure 8A are repeated at different training epochs using synapses trained under reduced multisensory experience and results are shown in Figure 8B (simulations of the McGurk effect for the other two perturbed developments are presented in the Supplementary Material). Reduced multisensory exposures lead to fewer McGurk illusions. At 2,500 training epochs (~5-years), the percentage of correct auditory phonemes is still more than 65% vs. ~50% in the basal condition, and then reduced to 50% at 5,000 training epochs (10-years of age) vs. ~33% in the basal condition. A greater McGurk effect appears only at the end of the training period (correct auditory phoneme recognition less than 45%, vs. ~25% in the basal condition). This aligns well with experimental findings in ASD children, who generally show reduced susceptibility to the McGurk effect compared with their TD counterparts (Taylor et al., 2010; Irwin et al., 2011; Bebko et al., 2014; Stevenson et al., 2014b).

## Discussion

The present work was designed with two fundamental goals in mind: to realize a model of audio-visual speech integration and its maturation in neurotypical individuals that can provide a preliminary account of several sets of empirical data in the literature; and to investigate the possible origins of differences in audiovisual perception that have been observed in children with an ASD.

It is well known that congruent visual articulatory information enhances an observer's speech perception abilities, improving the number of correct identifications (Sumby and Pollack, 1954; Calvert et al., 1998), and, conversely, that observation of incongruent speech gestures can rather dramatically alter what is perceived (McGurk and MacDonald, 1976). Traditionally, these multisensory interactions were considered to occur in high-level heteromodal association cortices (such as the pSTG/S). In recent years, however, several studies showed that integration can also occur at very early stages of cortical processing in regions that were traditionally considered to be unisensory (Foxe et al., 2000; Falchier et al., 2002, 2010; Molholm et al., 2002; Foxe and Schroeder, 2005; Smiley and Falchier, 2009; Molholm and Foxe, 2010).

The present model, in its mature architecture, effectively incorporates both mechanisms. The network realizes an adult configuration in which a two-step process of multisensory integration is implemented. While this is surely highly simplified given the extensive nature of MSI that is known to occur throughout subcortical and cortical structures, we submit that it represents the essential processes involved in the development of audiovisual multisensory speech perception. To recapitulate, the first process is at the level of unisensory areas where speech features are represented, and MSI occurs due to the presence of cross-sensory connections between visual and auditory neurons coding for the same (or similar) speech units. The second is at the level of a higher-order multisensory area known to be involved in multisensory speech processing, and here MSI is due to the presence of convergent feedforward connections from the aforementioned unisensory areas.

An important feature of the model is its capacity to mimic the increase in MSI performance that is observed across childhood development. The model assumes that connections are plastic and can be trained using Hebbian mechanisms of connection potentiation and depression. The latter aspect is not only useful to understand how multisensory speech recognition capabilities may improve with age, but can also contribute to understanding differences between neurotypical children and children with an ASD. Training parameters were assigned to simulate results of a recent study by our group that investigated audio-visual speech recognition abilities in TD children (Foxe et al., 2015). Several aspects of our maturation results in the TD configuration deserve attention:

- Performance in response to auditory unisensory stimuli improves significantly during the first epochs of training (first 2,000–2,500 epochs, corresponding to an age approximately below 5 years). Subsequently, improvement becomes very modest and almost completely ceases above 4,500–5,000 epochs (approximately 9–10 years). The model explains this behavior via a quick reinforcement of auditory feedforward connections, as a consequence of a 100% presence of the auditory input, and with the presence of an upper saturation for connection strength.
- Performance in response to visual unisensory input remains quite modest throughout the training period, but improves progressively above 2,000 epochs (4 years). This is a consequence of the smaller percentage of visual inputs used during training.
- For multisensory stimulation, gain (AV-A) improves significantly until approximately 6,500 epochs (13 years of age), reflecting the two mechanisms: the progressive improvement of the feedforward visual connections, and the strengthening of the cross-sensory connections between the two unisensory areas. Hence, the Hebbian mechanisms of learning can fully account for the maturation of multisensory speech perception observed in behavioral studies.

Moreover, with the mature TD configuration, the model could account for several additional results. We simulated the temporal pattern of previously observed cortical responses quite well, in the presence of both auditory and audio-visual speech inputs (Besle et al., 2008); we mimicked the decrease in the latency of the response (about 10 ms) observed in electrophysiological data in the presence of combined audio-visual speech stimuli compared with the auditory input only (Arnal et al., 2009); and finally, we simulated the main aspects of the McGurk effect.

It is worth noting that whereas in the present instantiation of the model we did not introduce feedback from the multisensory region to the unisensory areas, in order to minimize model complexity, inclusion of a feedback mechanism may be required to simulate additional aspects of the data (for instance, the presence of delayed cortical responses, see Arnal et al., 2009). This point may be the subject of future model extensions (see section Discussion below).

Since the mechanism underlying decreased efficacy of MSI in ASD remains in question, the second part of this work was committed to identifying what perturbations of the system provided the best fit for the observed ASD data. We used the model to test three alternative scenarios: a reduced number of multisensory exposures simulating reduced attention/exposures to facial gestures; a reduced learning factor for the reinforcement of connections in the Hebbian rule; and finally, a decreased number of synaptic connections among the regions in the network.

In the last two scenarios, the mature network abilities for speech recognition disagreed with previous empirical findings from Foxe et al. (2015). In the case of impaired connectivity, the simulations showed very poor performance for both unisensory and multisensory stimuli. Auditory stimuli, presented alone, reached a correct detection rate of just 25% at the end of training, compared to the 35% correct recognition rate seen in the experimental results. Similarly, cross-modal stimulations reached a peak of about 45% correct recognition in the final architectural configuration, which was far from simulating the behavioral data (more than 60% correct recognition rate).

In the case of a lower learning factor, the developmental profile of speech MSI abilities was similar to that observed in ASD children: in both cases, there was delayed maturation of correct speech-detection followed by a linear improvement, which reached TD-like abilities during the final epoch of training (simulating the 17-year-old population). Problematic here is the similar profile shown for auditory-alone and visual-alone speech perception. These results suggest that impaired learning would affect not only the acquisition of the MSI capabilities, but also the maturation of unisensory abilities, a result not seen in the empirical data of Foxe et al. (2015).

Conversely, the first scenario, a reduced proportion of multisensory experience, is the only one that produced results comparable with the empirical data. The notion of reduced attention to faces during speech perception in individuals with autism finds support in the literature. For example, studies have shown that infants at risk for ASD and children with a diagnosis of ASD pay less attention to a speaker's face (see for instance, Guiraud et al., 2012; Grossman et al., 2015), and that toddlers with a diagnosis of autism have a reduced bias toward faces in comparison to typically developing controls (Klin et al., 2009; Chawarska et al., 2010). Clearly such behavioral tendencies would lead to reduced multisensory speech experiences during development. This tendency may be overcome in later adolescence/adulthood by the extensive intervention that individuals with ASD often receive, and/or due to shifting priorities in adolescence.

To simulate reduced attention to multisensory experience here, during training a higher proportion of auditory-only stimuli were presented as compared to audiovisual stimuli. Due to the Hebbian rule used to train connections, the relative lack of multisensory experiences greatly affected the maturation of the connections, both between the unisensory regions (cross-sensory connections) and from the visual region targeting the multisensory elements. As a result, there was poorer capacity to recognize visual gestures (see Figure 7, second panel) and poorer performance when stimulated with a multisensory input. Conversely, auditory capacity was almost equal in the TD and ASD groups. These MSI differences, however, were significantly attenuated at the end of training.

These results agree very well with observations in Foxe et al. (2015) (see Figures 4, 7) and with other data in the literature. In particular, Smith and Bennetto (2007) observed that, in auditory only conditions, individuals with autism exhibited a similar threshold of speech-to-noise ratio (at 50% word recognition) compared with TD controls. Conversely, with the addition of visual information, the group with autism showed a smaller improvement in performance compared with controls. Furthermore, these authors observed reduced lip-reading capacity in their ASD group. All these behavioral data substantially agree with our model results (see Figures 4, 7).

As an additional consequence of weaker connectivity between the visual and multisensory area following reduced multisensory experiences, and ensuing reduced cross-sensory connectivity, the model showed fewer McGurk illusions, a result that has been consistently reported in previous studies on autism (Smith and Bennetto, 2007; Mongillo et al., 2008; Taylor et al., 2010; Irwin et al., 2011; Bebko et al., 2014; Stevenson et al., 2014b) and that finds indirect support from fMRI data from Nath and Beauchamp (2012). These authors found that the level of activity in pSTG/S was correlated with the likelihood of the McGurk effect. The same is present in this model where the highest probability of McGurk effect appears at the end of the training condition with high multisensory experience. This produces the strongest connectivity in the network, and leads to the highest activity in pSTG/S in cases of AV stimulation. Vice versa, in cases of training with reduced multisensory experience, the network presents less effective connections, and this leads to lower activity elicited in the pSTG/S region during multisensory stimulation.

One might ask how MSI in ASD compares with MSI function in individuals who are born functionally blind (i.e., deprived of one sense) but later recover visual function. Work from Roder et al. (Putzar et al., 2010b; Guerreiro et al., 2015, 2016) suggests that for individuals with congenital cataracts (CC patients) that are subsequently removed within the first 2 years, there is atypical development of MSI. For these individuals it has been shown that even when typical visual-only identification of the visual component of McGurk syllables is seen in adulthood (a subset of the participants studied), there is reduced susceptibility to the McGurk effect. In contrast, in autism there is recovery of MSI in adulthood in ASD for speech stimuli. MSI deficits for speech in childhood but not adulthood are seen not only in our data, but also in studies looking at the McGurk effect (Taylor et al., 2010; Irwin et al., 2011; Bebko et al., 2014; Stevenson et al., 2014b). This difference may reflect that, whereas in congenital cataract patients there is a period of complete visual deprivation, such a period is not present in autism. Consequently, cataract patients may undergo long-lasting reorganization within the visual cortex (Putzar et al., 2010a; Guerreiro et al., 2016) that impacts MSI. Therefore, the maturation of MSI in ASD patients and in cataract patients may be affected by different phenomena, mainly reduced attention to visual stimuli in ASD (as suggested by our model), but altered connectivity in the visual circuitry in sight-recovered humans. Obviously this is highly speculative and remains to be specifically tested.

## Other models of multisensory speech perception, and future directions

We should point out that a number of computational models have been developed in recent years to investigate the general problem of multisensory integration in the brain (Cuppini et al., 2011a; Ursino et al., 2014). Some of these models assume that integration is an emergent property based on network dynamics (Patton and Anastasio, 2003; Magosso et al., 2008; Ursino et al., 2009). Others have been realized to deal with the problem of multisensory integration in semantic memory and to link semantic content with lexical aspects of language (Rogers et al., 2004; Ursino et al., 2010, 2011, 2013, 2015). But the majority of these computational efforts to tackle multisensory integrative abilities and their maturation in the brain are based on a Bayesian approach (Anastasio et al., 2000; Ernst and Banks, 2002; Alais and Burr, 2004; Knill and Pouget, 2004; Shams et al., 2005; Körding et al., 2007; Rowland et al., 2007) in agreement with several psychophysical studies, showing that human behavior in a variety of tasks is nearly Bayes-optimal (Battaglia et al., 2003; Alais and Burr, 2004; Shams et al., 2005). Specifically for the problem of speech recognition, Ma and colleagues implemented a Bayesian model of optimal cue integration that could explain visual influences on auditory perception in a noisy environment, in agreement with experimental evidence (Ma et al., 2009). They analyzed the role of signal reliability in the formation of the multisensory likelihood function, and explained different experimental behaviors in the multisensory perception of words based on their representation as a collection of phonetic features in a topographically organized feature space.

Although it is quite clear that multisensory integration operates quasi-optimally when dealing with stimulus uncertainty, and despite all these efforts, very little is still known about the neural mechanisms engaged in this optimality. In particular, none of the previous computational models explained either the maturation of integrative abilities in speech perception or the different developmental trajectory for ASD, and how these capabilities might be instantiated in neural circuitry.

The present model is built to overcome these limits. It suggests a possible neural implementation of multisensory integration in speech perception that accounts for different experimental findings, without a direct connection with Bayesian inference. Moreover, this network is able to account for the experimental evidence of the differences in speech-detection performance in ASD subjects and the maturation of these processes in ASD over the course of development. It will be very interesting in future work to analyze which aspects of the model may be reconciled with Bayesian models and attempt to fuse the two approaches into a single model.

Finally, we wish to point out some lines for future investigation. First, in the present version of the model, the unisensory speech events are described by the same mathematical implementation and they are differentiated only by their position in unisensory regions. Thus, we simulated them all as equally detectable. In future versions of the model, we can make use of more detailed and biologically realistic descriptions of unisensory auditory and visual representations of words, for instance describing them as collections of sensory features, as in Ma et al. (2009) and other recent computational representations of sematic/lexical memory (Rogers et al., 2004; Ursino et al., 2010, 2013, 2015). In this way, one might better account for the correlation between speech events (i.e., events having some common or similar features) and simulate their differential detectability.

Another improvement may include the presence of feedback connections from the multisensory region to the unisensory ones. Such feedback may be especially important to describe model behavior over a longer temporal window: in our model the multisensory region is active after about 60–150 ms from the onset of auditory stimulation, a time in agreement with several experimental results (see for instance, Besle et al., 2008; Arnal et al., 2009; Brandwein et al., 2013). Hence, the effect of feedback from the multisensory to the unisensory areas should be apparent (assuming a 15 ms time constant and a 50 ms time delay for the feedback, as in the feedforward connections, see Table 1 in the Supplementary Material) by about 130 ms or later; this may be useful to analyse the late aspects of the observed responses in the aforementioned work.

An important challenge for a future analysis will be to reconcile the present model results with results concerning temporal acuity, not manipulated here (see for example van Wassenhove et al., 2007). Impairments in temporal processing are well documented in ASDs, indicating that individuals with ASD may have a larger audiovisual temporal binding window, i.e., they tend to perceive highly asynchronous stimuli as synchronous, hence as originating from the same event (Brock et al., 2002; Stevenson et al., 2014a). Moreover, the temporal binding window continues to decrease across development, even in TD individuals, and is smaller in adults than in children and adolescents (Hillock-Dunn and Wallace, 2012). Finally, the width of the temporal binding window seems to be inversely correlated with the McGurk effect (Wallace and Stevenson, 2014).

## Conclusion

(1) The simple architecture of the model, with 2 main mechanisms, cross-modal and feedforward connections, is able to explain and account for the maturation of speech-perception abilities. Reinforcement of feedforward connections is responsible for the quick acquisition of mature unisensory speech-perception, but it also represents the first step for the attainment of effective multisensory integrative abilities. This is fully accomplished only thanks to the concurrent strengthening of cross-sensory connections that produces the complete maturation of speech MSI. (2) The model was used to explore possible specific impairments responsible for the different developmental trajectory in children with an ASD, and it not only pointed at the more appropriate one, but it also discounted two alternate possibilities. As such, the model has been helpful in clarifying what accounts for multisensory speech integration deficits in ASD, and suggests possible training strategies to improve the development of multisensory speech processing in this population. Among the different hypotheses tested with this network, different attention/exposures to facial gestures provided the best fit for the observed empirical data on differences between typically developing children and children with an ASD. (3) The same architecture underlying the acquisition of MSI can support/explain other integrative phenomena: such as speeded RTs under conditions of multisensory stimulation and susceptibility to multisensory speech illusions (i.e., the McGurk effect).

## Author contributions

CC implemented the model. CC, SM, and MU analyzed the results. CC, SM, JF, MU, EM, and LR wrote the manuscript.

### Conflict of interest statement

The authors declare that the research was conducted in the absence of any commercial or financial relationships that could be construed as a potential conflict of interest.
