# Supplementary material for: A Computational Analysis of Neural Mechanisms Underlying the Maturation of Multisensory Speech Integration in Neurotypical Children and Those on the Autism Spectrum
Source: Front Hum Neurosci. 2017 Oct 30;11:518. doi: 10.3389/fnhum.2017.00518 (PMC5670153; doi:10.3389/fnhum.2017.00518)
Supplement: Supplementary file 1 [file DataSheet1.docx]

**Appendix**

**The Basal Model: mathematical description**

Each neuron will be denoted with a superscript, *m* or *n*, referred to a specific cortical area (*m, n* = *a*, *v, s,* for the auditory, visual, or multisensory region), and a subscript, *j* or *k*, which indicates the position within that area (*j*, *k* = 1,2,…*N*; here we arbitrarily chose N = 100). *u(t)* and *y(t)* are used to represent the net input and output of a given neuron at time *t*. Thus, $y_{j}^{m}(t)$ represents the output of a unit at position *j*, belonging to the area *m*, described by the following differential equation:

$\tau\frac{dy_{j}^{m}(t)}{dt}=-y_{j}^{m}(t)+F\left(u_{j}^{m}(t)\right)$ (1)

where *τ * is the neuron time constant and *F*(*u*) represents a sigmoidal relationship:

$F(u_{j}^{m})=\frac{1}{1+e^{-s(u_{j}^{m}-\theta)}}$ (2)

*s* and *θ* are parameters which establish the slope and the central position of the sigmoidal relationship, respectively. The saturation value was set at 1, i.e., all activities were normalized to the maximum.

For simplicity, neurons belonging to the three regions are described by the same parameters and the same time constant.

The net input reaching a neuron (i.e., the quantity $u_{j}^{m}(t)$ in Eq. 1) is the sum of two terms: a *within-region component* (say $l_{j}^{m}(t)$), due to the lateral connections from other neurons in the same area, and a component coming from *extra-area sources* (say $o_{j}^{m}(t)$). Hence

$u_{j}^{m}(t)=l_{j}^{m}(t)+o_{j}^{m}(t);$ (3)

To simulate the lateral input, $l_{j}^{m}(t)$, neurons within each area interact via excitatory and inhibitory connections: each neuron excites (and is excited by) its proximal neurons, and inhibits (and is inhibited by) more distal neurons. Thus, the input that a unisensory neuron receives from other elements of the same area is defined as:

$l_{j}^{m}(t)=\sum_{k} L_{jk}^{m}\cdot y_{k}^{m}(t);$ (4)

$L_{jk}^{m}$ is the strength of the lateral connection from a presynaptic neuron at position *k* to a postsynaptic neuron at position *j* both belonging to the same region *m,* and$y_{k}^{m}(t)$ is the activity of the presynaptic neuron at position *k*. These connections are symmetrical and arranged according to a “Mexican hat” distribution (a central excitatory zone surrounded by an inhibitory annulus):

$L_{jk}^{m}=\left\{\begin{matrix} \text{L}_{\text{ex0}}\cdot\text{e}^{-\frac{\text{(d}_{\text{jk}}\text{)}^{2}}{\text{2σ}_{\text{ex}}^{2}}}-\text{L}_{\text{in0}}\cdot\text{e}^{-\frac{\text{(d}_{\text{jk}}\text{)}^{2}}{\text{2σ}_{\text{in}}^{2}}}\text{ if d}_{\text{jk}}\neq0; \\ \text{0 if d}_{\text{jk}}=0; \end{matrix}\right.$ (5)

$L_{ex0}$ and $\sigma_{ex}$ define the excitatory Gaussian function, while $L_{in0}$ and $\sigma_{in}$ the inhibitory one, and $d_{jk}$ represents the distance between the pre-synaptic and post-synaptic neurons in the same area. To avoid undesired border effects, connections are realized by a circular structure so that every neuron of each area receives the same number of side connections. This is obtained assuming the following expression for the distance:

(6)

We assumed that both unisensory areas have the same pattern of lateral connections, in order to limit the number of hypotheses. Lateral connections in the multisensory region were set to improve the identification of the barycenter of the evoked activity.

The external component of the input, $o_{j}^{m}(t)$, represents all contributions from far positions (either the external world or other areas in the brain).

The external inputs have a different expression for the unisensory (*m* = *a*, *v*) and for the multisensory area (*m* = *s*).

The input to each *unisensory area* comprises a sensory stimulus from the external world (say $e_{j}^{m}(t)$), a cross-modal term from the other unisensory area (say $c_{j}^{m}(t)$), and a *noise component*, *n*, realized by a standard uniform distribution on an interval [−*n_max_* +*n_max_*], where *n_max_* is equal to the 30% of the strongest external stimulus used during the tasks; hence

$o_{j}^{m}(t)=e_{j}^{m}(t)+c_{j}^{m}(t)+n\text{ }m=a,v$ (7)

The first was simulated via a spatial Gaussian function, to reproduce the uncertainty in the detection of external stimuli. Assuming a stimulus (a simulated phoneme) coded in modality *m* (*m* = *a* or *v*) by a neuron at the position *p^m^* , the consequent input to the network can be written as

$e_{j}^{m}(t)=E_{0}^{m}e^{-\frac{(d_{j}^{m})^{2}}{2(\sigma_{m})^{2}}}$ (8)

$E_{0}^{m}$ represents the strength of the stimulus, $d_{j}^{m}$ is the distance between the neuron at position *j* and the stimulus at position *p^m^*, and $\sigma_{m}$ defines the degree of uncertainty in sensory detection (which results in the overall number of elements in region *m*, activated by the same external stimulus). $E_{0}^{m}$ can vary from simulation to simulation up to the 40% of its value to mimic the perceptual differences among subjects, and from task to task. This variability is computed from a uniform distribution. As previously described for the lateral connections, also the external inputs were implemented as having a circular structure; hence, the distance *d_j_* is defined as:

(9)

Finally, the cross-modal input, $c_{j}^{m}(t)$, was obtained assuming that each neuron received an excitation from the neurons of the other modality:

$\begin{matrix} c_{j}^{a}(t)=\sum_{k=1}^{N} W_{jk}^{av}\cdot y_{k}^{v}(t-\Delta t^{c}) \\ c_{j}^{v}(t)=\sum_{k=1}^{N} W_{jk}^{va}\cdot y_{k}^{a}(t-\Delta t^{c}) \end{matrix}$ (10)

The weights $W_{ij}^{av},W_{ij}^{va}$ were subject to a training phase. In the immature configuration, these connections were assumed ineffective. $\Delta t^{c}$ is a delay representing the latency of propagation between the two cortices.

The excitatory external input to the multisensory neurons is due to the feedforward connections from the unisensory areas. Hence

$i_{j}^{s}(t)=\sum_{k=1}^{N} W_{jk}^{sa}\cdot y_{k}^{a}(t-\Delta t^{s})+\sum_{k=1}^{N} W_{jk}^{sv}\cdot y_{k}^{v}(t-\Delta t^{s})$ (11)

$W_{jk}^{sa}$and $W_{jk}^{sv}$ are the connections linking the presynaptic neuron at position k in the unisensory area (auditory and visual, respectively) to the jth neuron in the multisensory area, and $\Delta t^{s}$ is the delay from the unisensory to the multisensory region. These connections were subject to a training phase too. We chose a simple 1-to-1 connectivity to set their immature configuration:

$W_{jk}^{sm}=\left\{\begin{matrix} W_{0}^{sm},\text{ if j}=\text{k} \\ 0,\text{ if j}\neq\text{k} \end{matrix}\right.\text{ }m=a,v$ (12)

$W_{0}^{sm}$ is the initial synaptic efficacy. In the model, we set $W_{0}^{sa}=W_{0}^{sv}$to minimize the simplifying hypotheses of the model.

**Training the network**

During this phase visual and auditory stimuli are presented to the network, alone or congruently combined (i.e. corresponding the same phoneme representations in the two modalities), and inter-area connections (i.e., connections $W_{jk}^{av}$and $W_{jk}^{va}$ in Eq. 10, and connections $W_{jk}^{sa}$and $W_{jk}^{sv}$ in Eq. 11-12) are trained with a Hebbian rule. The immature configuration was given to simulate an initial poor capacity to detect phonemes. The training modifies the synaptic weight based on the correlation between the presynaptic and postsynaptic activity via the following training rule:

(13)

which is an Hebb rule with learning rate $\gamma^{mn}$ and a decay term depending on the actual strength of the synapsis $W_{jk}^{mn}$ and the highest value fixed for the synaptic reinforcement $W_{\max}^{mn}$ (see ([Hertz et al., 1991](#_ENREF_40)), and γ represents the learning factors. In particular, according to Eq. (13) at steady state we have:

E{} is the expected value. Since the activities of neurons are normalized between 0 and 1, the previous equation signifies that each connection cannot overcome the maximum value $\Delta W_{\max}^{mn}$ (that occurs when the presynaptic activity is close to 1, in 100% of cases).

According to Eq. (13), a postsynaptic neuron, in region *m* and position *j*, with a high activity $y_{j}^{m}$ modifies its targeting connections $W_{jk}^{mn}$, shaping them based on the actual activity $y_{k}^{n}$ of the presynaptic elements, in region *n* and position *k*. Conversely, silent neurons with poor output activity do not appreciably modify their connections. Moreover, the maximum value fixed for each pairs of long-range excitatory connections is introduced to implement a saturation in the synaptic reinforcement. Thus connections linking two neurons in the unisensory regions (say *j,* *k*) are modified as follows :

$W_{jk}^{mn}\leftarrow W_{jk}^{mn}+\gamma^{mn}\text{ }y_{j}^{m}\left(y_{k}^{n}-\frac{W_{jk}^{mn}}{W_{\max}^{c}}\right)\text{ }$ *mn=va, av* (14)

with $\gamma^{va}=\gamma^{av}=\gamma^{c}$. The feedforward connections are modified according to a similar function, but with different learning rate and saturation value:

$W_{jk}^{sn}\leftarrow W_{jk}^{sn}+\gamma^{sn}\text{ }y_{j}^{s}\left(y_{k}^{n}-\frac{W_{jk}^{sn}}{W_{\max}^{s}}\right)\text{ }$ *n=v, a* (14’)

Also in this case the learning rate is fixed and equal for the two modalities: $\gamma^{sa}=\gamma^{sv}=\gamma^{s}$.

In Eq. 14 and 14’ the synaptic weights cannot overcome a maximum saturation value ($W_{Max}^{c}$ for connections $W_{jk}^{av}$and $W_{jk}^{va}$ and $W_{Max}^{s}$ for connections $W_{jk}^{sa}$and $W_{jk}^{sv}$).

**Table 1 – parameter values**

| **Neurons** | | **Connections** | |
| --- | --- | --- | --- |
| N = 100 | | = 2 | = 3 |
| *θ* = 18 | | = 1.8 | = 24 |
| *s* = 0.25 | | $W_{0}^{sv}$= 12 | $W_{0}^{sa}$= 12 |
| *τ*  = 15ms | | $\Delta t^{s}$ = 50ms | $\Delta t^{c}$ = 10ms |
| **Input** | | **Training phase** | |
| = 14 | = 2 | $\gamma^{c}$= 0.03 $\gamma^{s}$= 0.3 | |
| = [6 – 22] | = 2 | $W_{Max}^{c}$= 2 $W_{Max}^{s}$= 28 | |

**Parameter assignment**

The values of model parameters (see Table 1) were assigned in accordance with the criteria summarized below, based on findings reported in the literature. Parameters describing the elements of the network were assumed equal for the two modality-specific sensory regions, to reduce the number of ad hoc assumptions.

*External inputs* - During training, the strength and the width of the Gaussian functions (parameters $E_{0}^{v}$and $E_{0}^{a}$ and$\sigma^{a},\sigma^{v}$) were chosen so that: the overall input elicited a response in the upper portion of the linear part of the sigmoidal static characteristic. During the different set of simulations, as described in the method section, the amplitude of visual input, $E_{0}^{v}$, was chosen to generate an activity in pSTG/S area slightly below the recognition threshold; for the auditory stimuli, we used 7 different levels of input amplitude (parameter$E_{0}^{a}$). They ranged from ineffective (equal to 6, which minimally activates the auditory region and generates 0% correct identifications) to a maximum level (able to saturate the auditory evoked activity and generates more than 80% correct identification in the adult, equal to 22), with $\Delta E_{0}^{a}=2.5$. In each simulation, we kept fixed the noise added to all neurons to the 30% of the maximum level of $E_{0}^{a}$. These values allowed to mimic the different auditory signal-to-noise ratios used for speech recognition tasks in ([Foxe et al., 2015](#_ENREF_32)).

*Parameters of individual neurons* – The central abscissa, θ, was assigned to have negligible neural activity in basal conditions (i.e., when the input was zero). The slope of the sigmoidal relationship, s, was assigned to have a smooth transition from silence to saturation in response to external stimuli.

*Temporal aspects of neurons and synaptic connections* – Parameters which characterize the dynamics (time constants and pure latencies) were selected to reproduce the time patterns of neural activation reported in ([Besle et al., 2008](#_ENREF_12)), ([Arnal et al., 2009](#_ENREF_5)) and ([Brandwein et al., 2011](#_ENREF_16); [Brandwein et al., 2013](#_ENREF_14)).

In particular, the time delay of cross-sensory connections, $\Delta t^{c}$, was assumed as low as 10 ms, which agrees with the time delay observed by ([Besle et al., 2008](#_ENREF_12)) in which activity in auditory cortex in response to visual speech movements was delayed by 10 ms compared to activation of higher order visual area MT/V5. The time constant $\tau$ of the neurons was chosen in agreement with values (a few ms) normally used in deterministic mean-field equations ([Treves, 1993](#_ENREF_87); [Ben-Yishai et al., 1995](#_ENREF_10)) and so that the transient response of the auditory cortex peaks at about 65-70 ms from an auditory stimulus, as in ([Besle et al., 2008](#_ENREF_12)). This choice of time constants is also compatible with onset of unisensory cortical responses observed in ([Brandwein et al., 2013](#_ENREF_14)) in their oldest TD group.

The time delay of the feedforward connections, $\Delta t^{s}$, was chosen to mimic the temporal activation (peaking at about 120 ms) observed in multisensory pSTG/S by ([Besle et al., 2008](#_ENREF_12)) after an auditory input. These responses also agree with the spatiotemporal characteristics of audio-visual interactions reported by ([Brandwein et al., 2013](#_ENREF_14)) who observed significant audiovisual interactions between 100 and 130 ms in groups of children and adolescents with and without ASD.

*Strengths of synaptic connections* – Parameters which established the width and the strength of lateral connections (i.e., $L_{ex0}$, $L_{in0}$, $\sigma_{ex}$ and $\sigma_{in}$) were assigned to satisfy several criteria: (1) inhibition must be strong enough to warrant competition between two stimuli in the same area, and (2) the balance between excitation and inhibition must avoid instability, i.e., an uncontrolled excitation which propagates to the overall area.

The maximum value of inter-area synaptic strength ($W_{Max}^{c}$), between the unisensory regions was assigned to be high enough so that (3) the cross-sensory input from one area could reinforce response of neurons in the other area when these neurons are near or just above the activation threshold. However these connections are maintained sufficiently low so that (4) an external stimulus in one sensory modality did not induce phantom activity in the other sensory-specific area.

The initial value of the strength of connections targeting the multisensory region ($W_{0}^{sm}$) was set so that (5) an effective unisensory stimulus of sufficient strength (i.e., able to lead unisensory neurons close to saturation) evoked a weak activity (i.e., under the detection threshold) in the multisensory region. The maximum value of the strength of these connections ($W_{Max}^{s}$) was set so that (6) an effective unisensory stimulus of sufficient strength evoked a high activity in the multisensory region, after the training phase.

Finally, the ratio of the synaptic learning factors ($\gamma^{c}$and$\gamma^{s}$) was set so that the maturation of feedforward connections was quicker than the reinforcement of cross-sensory connections. This helped to simulate the quicker maturation of unisensory speech-recognition abilities with respect to the speech MSI. These four parameters and the sensory experience used to train the model (for A and AV: 2 parameters) were adjusted to achieve a good fit between the model and the data about the TD maturation as reported in ([Foxe et al., 2015](#_ENREF_32)). The values of these four parameters were not subsequently varied for the simulations testing the alternative explanations of impaired MSI in ASD.
