# Supplementary material for: A Computational Analysis of Neural Mechanisms Underlying the Maturation of Multisensory Speech Integration in Neurotypical Children and Those on the Autism Spectrum
Source: Front Hum Neurosci. 2017 Oct 30;11:518. doi: 10.3389/fnhum.2017.00518 (PMC5670153; doi:10.3389/fnhum.2017.00518)
Supplement: Supplementary file 2 [file Image1.PDF]

SUPPLEMENTARY MATERIAL

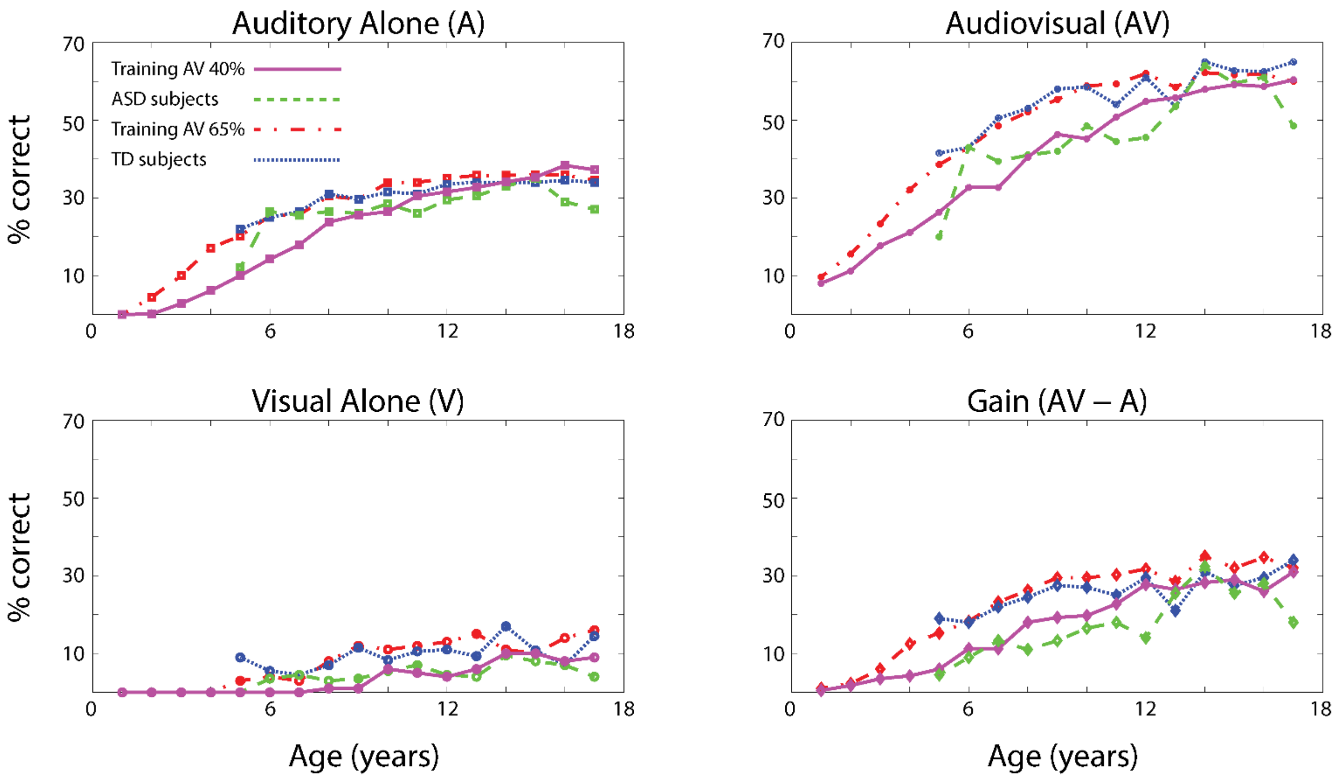

Figure S1 – Speech recognition performance in the auditory-alone (A), visual-alone (V), and audiovisual (AV) conditions, and AV gain (AV–V), in case of poor multisensory experience. *Average speech recognition performances (% correct) evaluated at different epochs during synaptic maturation for a subject with reduced multisensory experience simulating ASD (40% AV in its initial training condition — solid lines), compared with experimental data on ASD children during adolescence, as reported in Foxe et al. (Foxe et al., 2015) (dashed line). To improve comparison, the simulation results for typical subject (65% AV – simulating TD – dashed-dot lines) are also reported (these are the same simulations as in Figure 4) . The meaning of all panels is the same as in Figure 4 in the manuscript.*

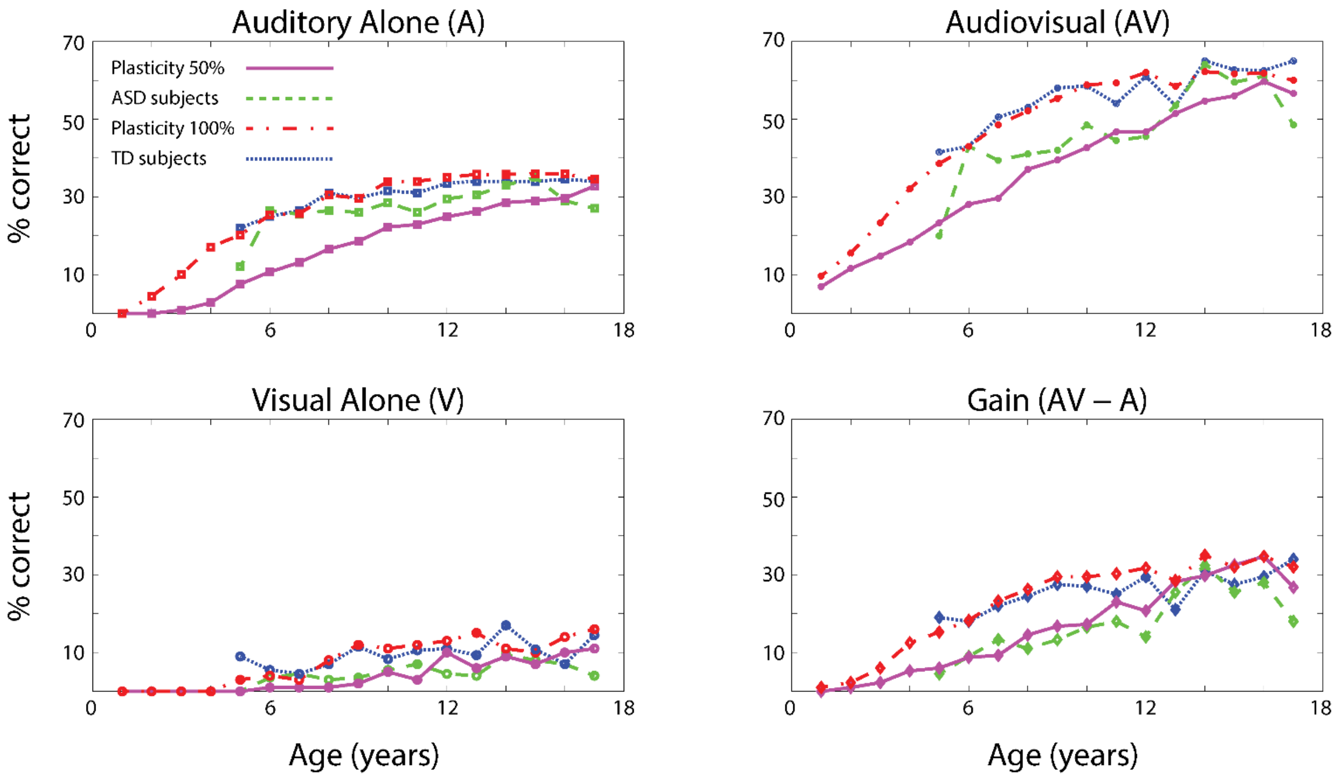

***Figure S2 – Maturation of speech recognition performance in the auditory-alone (A), visual-alone (V), and audiovisual (AV) conditions, and AV gain (AV–V), as a function of the synaptic plasticity**. Average speech recognition performance of the model (% correct) evaluated at different stages during training for a subject with reduced synaptic plasticity (50% - solid lines) simulating ASD. The network results are compared with experimental data from Foxe et al. (Foxe et al., 2015) where the speech-recognition tasks were evaluated at different ages (5 years – 17 years) during the adolescence of TD subjects (blue lines) and ASD (green lines). To improve comparison, the simulation results for typical subject (with a fully effective plasticity, simulating TD – dashed-dot lines) are also reported (these are the same simulations as in Figure 4). The meaning of all panels is the same as in Figure 4 in the manuscript.*

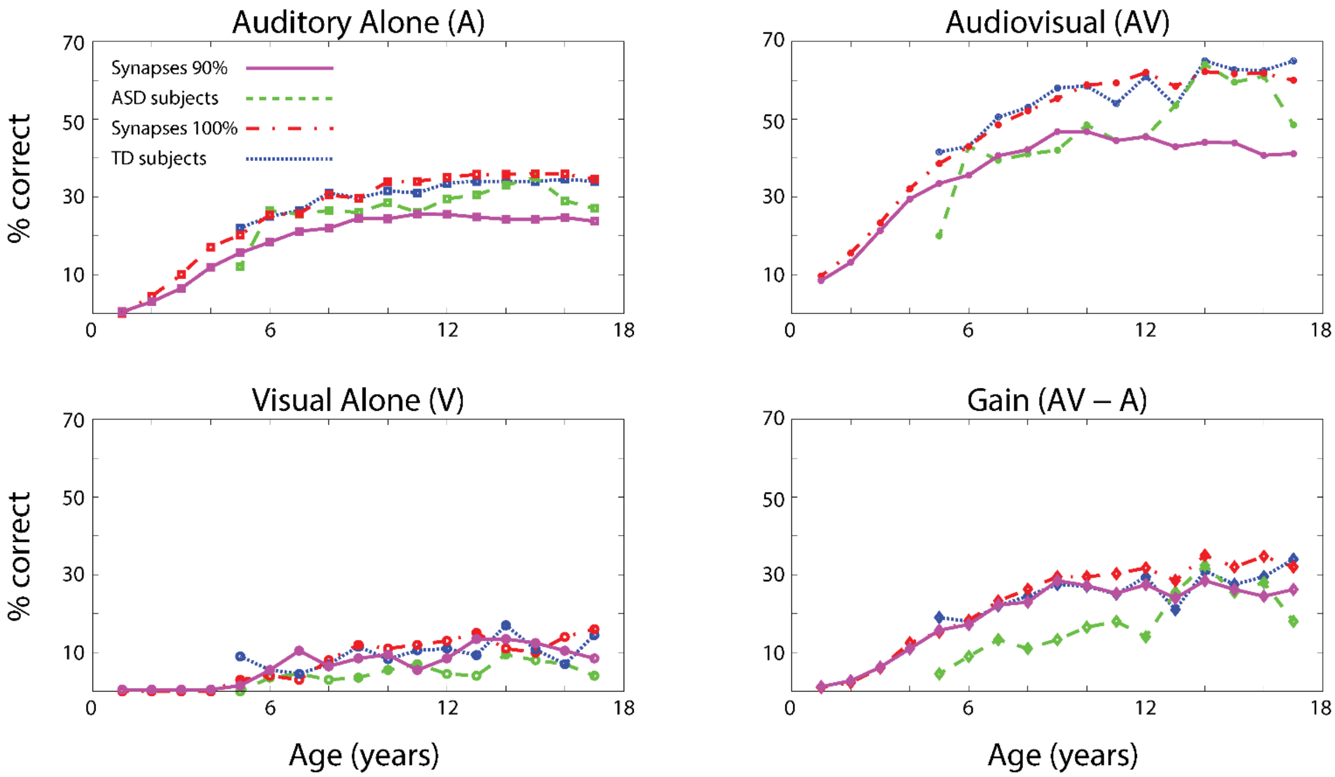

Figure S3 – Maturation of speech recognition performance in the auditory-alone (A), visual-alone (V), and audiovisual (AV) conditions, and AV gain (AV–V), as a function of the synaptic architecture. *Average speech recognition performance of the model (% correct) evaluated at different stages during training for a subject with reduced number of synaptic connections (90% - solid lines) simulating ASD. The network results are compared with experimental data from Foxe and colleagues (2015) where the speech-recognition tasks were evaluated at different ages (5 years – 17 years) during the adolescence of TD subjects (blue lines) and ASD (green lines). To improve comparison, the simulation results for typical subject (with fully effective connections, simulating TD – dashed-dot lines) are also reported (these are the same simulations as in Figure 4). The meaning of all panels is the same as in Figure 4 in the manuscript.*

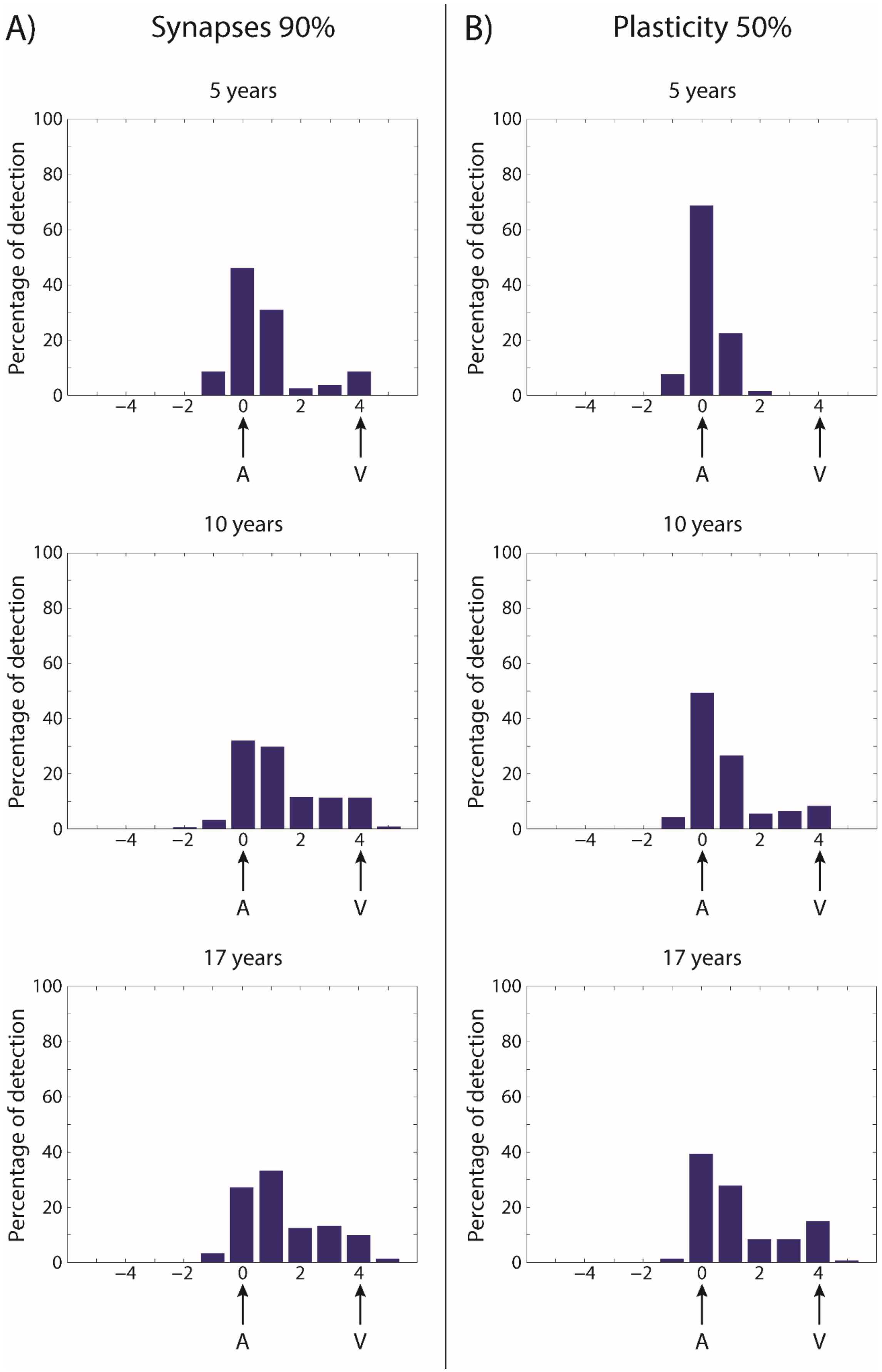
A)
Synapses 90%
B)
Plasticity 50%
5 years
10 years
17 years
Percentage of detection
100
80
60
40
20
0
−4
−2
0
2
4
A
V

Figure S4 – A) McGurk effect in case of reduced number of synaptic connections*. Results of simulations with incongruent visual and auditory stimuli (the same inputs as in Figure 8 of the manuscript), in case of a training condition with a reduced number of synaptic connections. The network resulted susceptible to the McGurk illusion as well as the simulated TD condition (intact network); that is, even after 5000 training epochs (corresponding to 10 years, Middle panel), the model is characterized by a strong visual influence on the auditory percept, displaying a strong McGurk effect (percentage of correct phoneme detection as low as 33%), although not as strong as in its final configuration. In this last configuration, the model presents the McGurk effect in almost 70% of cases.* ***B) McGurk effect in case of reduced synaptic plasticity****. Results of simulations with incongruent visual and auditory stimuli (the same inputs as in Figure 8 of the manuscript), in case of a training condition with a reduced plasticity of synaptic connections. The network resulted less susceptible to the McGurk illusion than the simulated TD condition. The network, even after 5000 training epochs (corresponding to 10 years, Middle panel), is characterized by a poor visual influence on the auditory percept; the correct auditory phoneme was still recognized in more than 50% of the cases. Only at the end of its maturation (Lower panel), the network presents a greater McGurk effect, even if it is not yet comparable with that in the simulated TD condition (correct auditory phoneme recognition in 40% of the simulations).*
